# Supplementary material for: Sex differences in risk factors for incident peripheral artery disease hospitalisation or death: Cohort study of UK Biobank participants
Source: PLoS One. 2023 Oct 18;18(10):e0292083. doi: 10.1371/journal.pone.0292083 (PMC10584119; doi:10.1371/journal.pone.0292083)
Supplement: S1 Checklist — (DOCX) [file pone.0292083.s001.docx]

STROBE Statement—checklist of items that should be included in reports of observational studies

|  | Item No. | Recommendation | Page  No. | Relevant text from manuscript |
| --- | --- | --- | --- | --- |
| **Title and abstract** | 1 | (*a*) Indicate the study’s design with a commonly used term in the title or the abstract | 1 | “cohort study”. |
|  |  | (*b*) Provide in the abstract an informative and balanced summary of what was done and what was found | 2 | Under “Methods” and “Results” of the Abstract. |
| Introduction | | | |  |
| Background/rationale | 2 | Explain the scientific background and rationale for the investigation being reported | 3 | “Although some studies have suggested that sex differences exist in risk factors for PAD, such as smoking, hypertension, diabetes, and C-reactive protein (CRP) [1, 7, 8], the extent of these differences remains to be reliably quantified.” |
| Objectives | 3 | State specific objectives, including any prespecified hypotheses | 3 | “In the current study, we used data from the UK Biobank to examine whether there are sex differences in the associations between major risk factors and incident PAD hospitalisation or death, and we also investigated whether any sex differences varied across major subpopulations.” |
| Methods | | | |  |
| Study design | 4 | Present key elements of study design early in the paper | 3-4 | “The UK Biobank is a large prospective cohort study of around half a million participants aged 40 to 69 years old recruited between 2006 and 2010.” “Participants attended one of 22 centres for baseline assessment, where written informed consent was obtained. Touchscreen questionnaires and nurse-led interviews were conducted to collect information on lifestyle and medical history, and physical and functional measurements were taken.” “Participants` baseline data were linked with hospital admission data from England, Scotland, and Wales and the national death register (primary or secondary cause of death) to identify the date of the first record of PAD.” |
| Setting | 5 | Describe the setting, locations, and relevant dates, including periods of recruitment, exposure, follow-up, and data collection |  |  |
| Participants | 6 | (*a*) *Cohort study*—Give the eligibility criteria, and the sources and methods of selection of participants. Describe methods of follow-up  *Case-control study*—Give the eligibility criteria, and the sources and methods of case ascertainment and control selection. Give the rationale for the choice of cases and controls  *Cross-sectional study*—Give the eligibility criteria, and the sources and methods of selection of participants | 4 | “Participants` baseline data were linked with hospital admission data from England, Scotland, and Wales and the national death register (primary or secondary cause of death) to identify the date of the first record of PAD. Participants who had a hospital admission(s) with PAD before baseline assessment were excluded.” |
|  |  | (*b*) *Cohort study*—For matched studies, give matching criteria and number of exposed and unexposed  *Case-control study*—For matched studies, give matching criteria and the number of controls per case | N/A | N/A |
| Variables | 7 | Clearly define all outcomes, exposures, predictors, potential confounders, and effect modifiers. Give diagnostic criteria, if applicable | Table 1  6 | Table 1  “Incident PAD documentation, from baseline to 30 September 2021, was the study endpoint.” |
| Data sources/ measurement | 8* | For each variable of interest, give sources of data and details of methods of assessment (measurement). Describe comparability of assessment methods if there is more than one group | Table 2 | Baseline characteristics of women and men were reported in Table 2. |
| Bias | 9 | Describe any efforts to address potential sources of bias | Table 1 | Potential confounders adjusted in multivariable analyses were listed in Table 1, column “Adjusted variables”. |
| Study size | 10 | Explain how the study size was arrived at | N/A | N/A |

| Quantitative variables | 11 | Explain how quantitative variables were handled in the analyses. If applicable, describe which groupings were chosen and why | Table 1 | Table 1, column “Definitions, measurements, and/or categorises”. |
| --- | --- | --- | --- | --- |
| Statistical methods | 12 | (*a*) Describe all statistical methods, including those used to control for confounding | 7 | “Baseline characteristics are presented as number (percentage) for categorical variables and as mean (standard deviation (SD)) or median (the first and third quartiles) for continuous variables as appropriate. Age-adjusted sex-specific incidence of PAD was modelled using Poisson regression. Cox proportional hazard regression models were used to estimate the hazard ratios (HRs) for each risk factor by sex, with interaction terms fitted between each risk factor and sex to obtain the women-to-men ratio of HRs (RHR) [16]. Each risk factor was initially adjusted only for age, then further adjusted for sets of covariates based on perceived probable causal relationships, determined a priori [9-11] (Table 1). We assessed the shape of the associations with the risk of PAD for continuous variables, using penalised smoothing splines, adjusted for the same set of covariates as in the Cox models.” |
|  |  | (*b*) Describe any methods used to examine subgroups and interactions | 7 | “We conducted subgroup analyses investigating whether sex differences in risk factors for PAD differed by age group (<60 and ≥60 years), by adding an interaction term between sex, age group, and the risk factor of interest to the models. Similarly, subgroup analyses were undertaken by smoking status (never versus ever), diabetes status, and SES (above versus below the UK national median (-0.56)). We also conducted subgroup analyses by antihypertensive medication (use versus not) for blood pressure measures, and by lipid-lowering medication for lipid profiles.” |
|  |  | (*c*) Explain how missing data were addressed | 7 | “Complete case analyses”. |
|  |  | (*d*) *Cohort study*—If applicable, explain how loss to follow-up was addressed  *Case-control study*—If applicable, explain how matching of cases and controls was addressed  *Cross-sectional study*—If applicable, describe analytical methods taking account of sampling strategy | N/A | N/A |
|  |  | (*e*) Describe any sensitivity analyses | 4 | “Since some adopted procedures might be conducted for aneurysms, they were only used in the absence of a diagnosis of aneurysms before or at the time of the procedure in a sensitivity analysis. In an additional sensitivity analysis, only diagnostic codes were used.” |
| Results | | | | |
| Participants | 13* | (a) Report numbers of individuals at each stage of study—eg numbers potentially eligible, examined for eligibility, confirmed eligible, included in the study, completing follow-up, and analysed | 8  8 | “After excluding 2206 participants (28.3% women, Additional file: Table S2) with a previous hospital admission for PAD at baseline, 500,207 individuals (54.5% women, mean age 56.5 years) were included in the analyses.” |
|  |  | (b) Give reasons for non-participation at each stage |  |  |
|  |  | (c) Consider use of a flow diagram | The exclusion was straightforward. Only those with a previous hospital admission for PAD at baseline were excluded. | |
| Descriptive data | 14* | (a) Give characteristics of study participants (eg demographic, clinical, social) and information on exposures and potential confounders | 8 | “The mean age was 56.3 years (SD 8) for women and 56.7 years (SD 8.2) for men (Table 2 and Additional file: Table S3).” |
|  |  | (b) Indicate number of participants with missing data for each variable of interest | 8 | “Percentages of missing for each variable were <7%, except for high-density lipoprotein cholesterol (HDL-C, 14.4%).” |
|  |  | (c) *Cohort study*—Summarise follow-up time (eg, average and total amount) | 9 | “Over 12.6 years (median) of follow-up”. |
| Outcome data | 15* | *Cohort study*—Report numbers of outcome events or summary measures over time | 9 | “7660 participants (34.7% women) had their first PAD documented (hospitalisation with PAD (n=7515) or PAD as the cause of death (n=145)).” |
|  |  | *Case-control study—*Report numbers in each exposure category, or summary measures of exposure |  |  |
|  |  | *Cross-sectional study—*Report numbers of outcome events or summary measures |  |  |
| Main results | 16 | (*a*) Give unadjusted estimates and, if applicable, confounder-adjusted estimates and their precision (eg, 95% confidence interval). Make clear which confounders were adjusted for and why they were included | Fig 1  S4 Table | Fig 1 for adjusted results.  S4 Table for age-adjusted results. |
|  |  | (*b*) Report category boundaries when continuous variables were categorized | Fig 1, Table 3, S4-12 Tables, S1-4 Figs | |
|  |  | (*c*) If relevant, consider translating estimates of relative risk into absolute risk for a meaningful time period | Table 3 | |

| Other analyses | 17 | Report other analyses done—eg analyses of subgroups and interactions, and sensitivity analyses | S5-6 Tables for sensitivity analyses.  S7-12 Tables for subgroups analyses. | |
| --- | --- | --- | --- | --- |
| Discussion | | | | |
| Key results | 18 | Summarise key results with reference to study objectives | 14-15 | “In the current study, among participants aged in a broad spectrum at baseline and followed up for over 10 years, the age-adjusted annual incidence of PAD hospitalisation or death was 79.6 and 185.3 per 100,000 population in women and men, respectively. This was comparable to the real-life age-adjusted data in Australia, where the rates in 2018-2019 (albeit being prevalent hospitalisation and not including death) were 77 and 152 per 100,000 population [17]. We assessed sex differences in a range of risk factors for PAD. Hypertension, type 1 and 2 diabetes, lower SES, lower kidney function, and higher CRP were each associated with a higher risk of PAD, similarly in both sexes. Being a current smoker, or having prior stroke or MI conferred 18%, 26%, or 50% higher excess risk of PAD, respectively, in women. Per 1 mmol/L higher HDL-C was associated with lower risk of PAD, which was 34% greater in women than in men.” |
| Limitations | 19 | Discuss limitations of the study, taking into account sources of potential bias or imprecision. Discuss both direction and magnitude of any potential bias | 19 | “There are several limitations worth noting. First, most UK Biobank participants are White, and are more likely to live in less socioeconomically deprived areas, and be healthier, than the general population [37]. Second, self-reported baseline information on smoking related variables might have introduced differential measurement errors by sexes. For instance, a recent Korean study found that cotinine (an objective measure of smoking inhalation) was higher in women than men at the same level of self-reported smoking [38], although this was not found in an earlier Scottish study [26]. Yet, although history of diabetes, stroke and MI, and use of antihypertensive and/or lipid lowering medication were also self-reported through touchscreen questionnaires, these were further confirmed in nurse-led interviews [39]. All the other variables were measured by the study team. Third, our assessment of PAD was based on hospitalisation or death. However, individuals with asymptomatic PAD (more common in women [4]) might not present to the healthcare system, and those who were on conservative management not requiring a procedure would not need hospitalisation. As a result, it is likely that we have missed PAD cases, which would lead to an underestimate of the real disease rates. The reported rates therefore represent those of PAD that is serious enough to cause hospitalisation or death. That said, PAD hospitalisations represent a substantial medical and financial burden for patients and healthcare system [40], and thus, are worth investigation. Finally, we note that multiple tests were conducted and the related concern of false positives [41]. Thus, the results should be interpreted in the light of the effect sizes and clinical relevance.” |
| Interpretation | 20 | Give a cautious overall interpretation of results considering objectives, limitations, multiplicity of analyses, results from similar studies, and other relevant evidence | 15-19 | We compared our results to relevant evidence for all the investigated risk factors. |
| Generalisability | 21 | Discuss the generalisability (external validity) of the study results | 19 | “First, most UK Biobank participants are White, and are more likely to live in less socioeconomically deprived areas, and be healthier, than the general population [37].” |
| Other information | |  | | |
| Funding | 22 | Give the source of funding and the role of the funders for the present study and, if applicable, for the original study on which the present article is based | Online submission form. | “This work is supported ‘GeorgeThink’ seed funding from The George Institute for  Global Health.” |

*Give information separately for cases and controls in case-control studies and, if applicable, for exposed and unexposed groups in cohort and cross-sectional studies.

**Note:** An Explanation and Elaboration article discusses each checklist item and gives methodological background and published examples of transparent reporting. The STROBE checklist is best used in conjunction with this article (freely available on the Web sites of PLoS Medicine at http://www.plosmedicine.org/, Annals of Internal Medicine at http://www.annals.org/, and Epidemiology at http://www.epidem.com/). Information on the STROBE Initiative is available at www.strobe-statement.org.
