# Supplementary material for: Sex differences in risk factors for incident peripheral artery disease hospitalisation or death: Cohort study of UK Biobank participants
Source: PLoS One. 2023 Oct 18;18(10):e0292083. doi: 10.1371/journal.pone.0292083 (PMC10584119; doi:10.1371/journal.pone.0292083)
Supplement: S1 Fig — (PDF) [file pone.0292083.s002.pdf]

(A) (B)

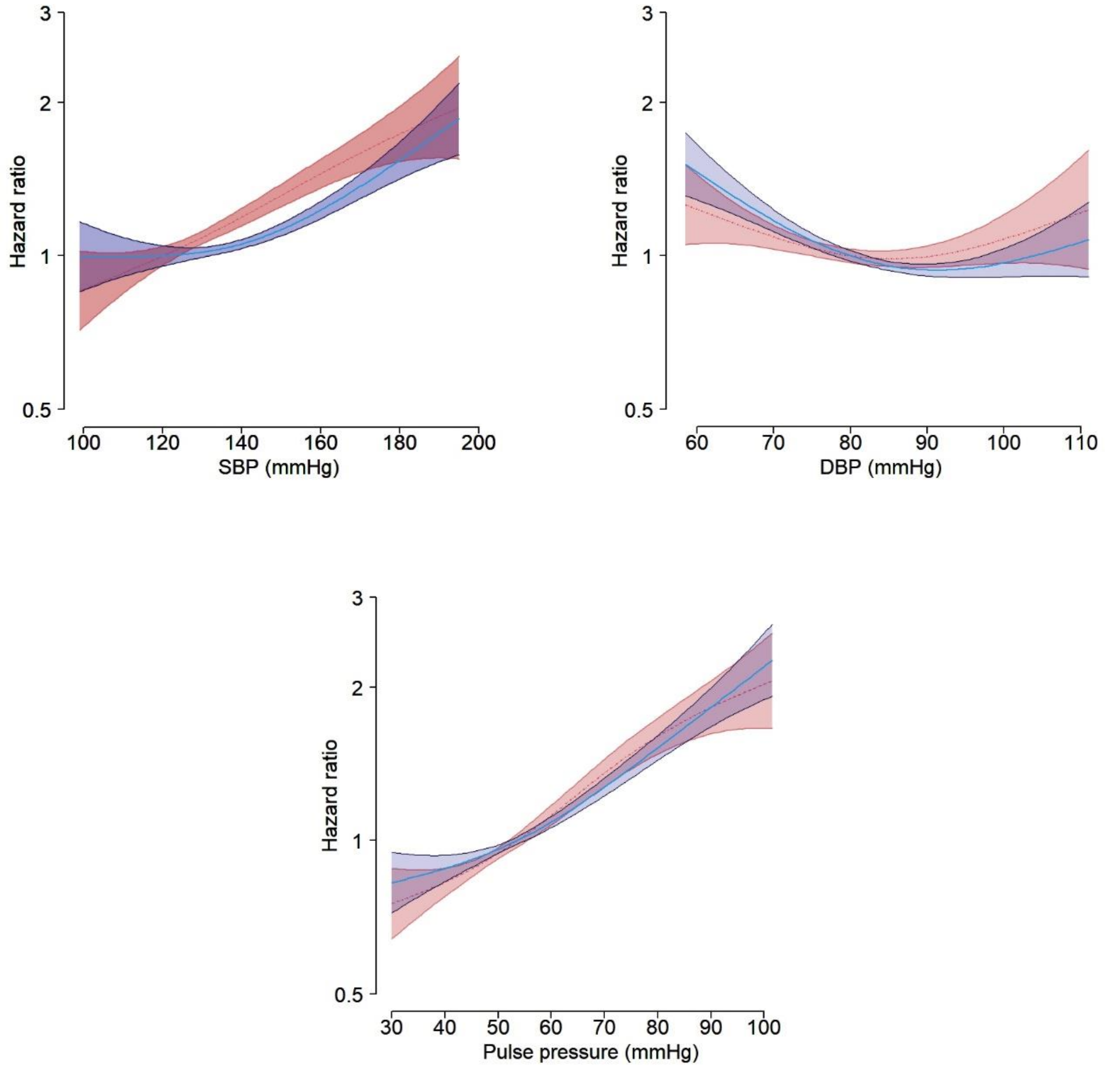

(C)

S1 Fig. Sex-specific multivariable-adjusted hazard ratios for blood pressure measures with the risk of peripheral artery disease.

DBP denotes diastolic blood pressure, SBP systolic blood pressure. Modelled with penalised smoothing splines, adjusted for age, diabetes, continuous measure of body mass index, total cholesterol, estimated glomerular filtration rate calculated using cystatin C, smoking, socioeconomic status, and lipid lowering and/or antihypertensive medications. Extreme values in the upper and lower 0.5% of the blood pressure distributions were excluded (ranges: SBP 99.0 to 195.0 mmHg, DBP 58.5 to 111.0 mmHg, and pulse pressure 30.0 to 101.5 mmHg). Reference value for SBP, DBP, and pulse pressure were 120.0 mmHg, 80.0 mmHg, and median value of 53.5 mmHg, respectively. The pink dotted lines represent the hazard function for women, and the pink shaded areas are the 95% confidence intervals for women. The blue lines represent the hazard function for men, and the blue shaded areas are the 95% confidence intervals for men.
