# Supplementary material for: Sex differences in risk factors for incident peripheral artery disease hospitalisation or death: Cohort study of UK Biobank participants
Source: PLoS One. 2023 Oct 18;18(10):e0292083. doi: 10.1371/journal.pone.0292083 (PMC10584119; doi:10.1371/journal.pone.0292083)
Supplement: S3 Fig — (PDF) [file pone.0292083.s004.pdf]

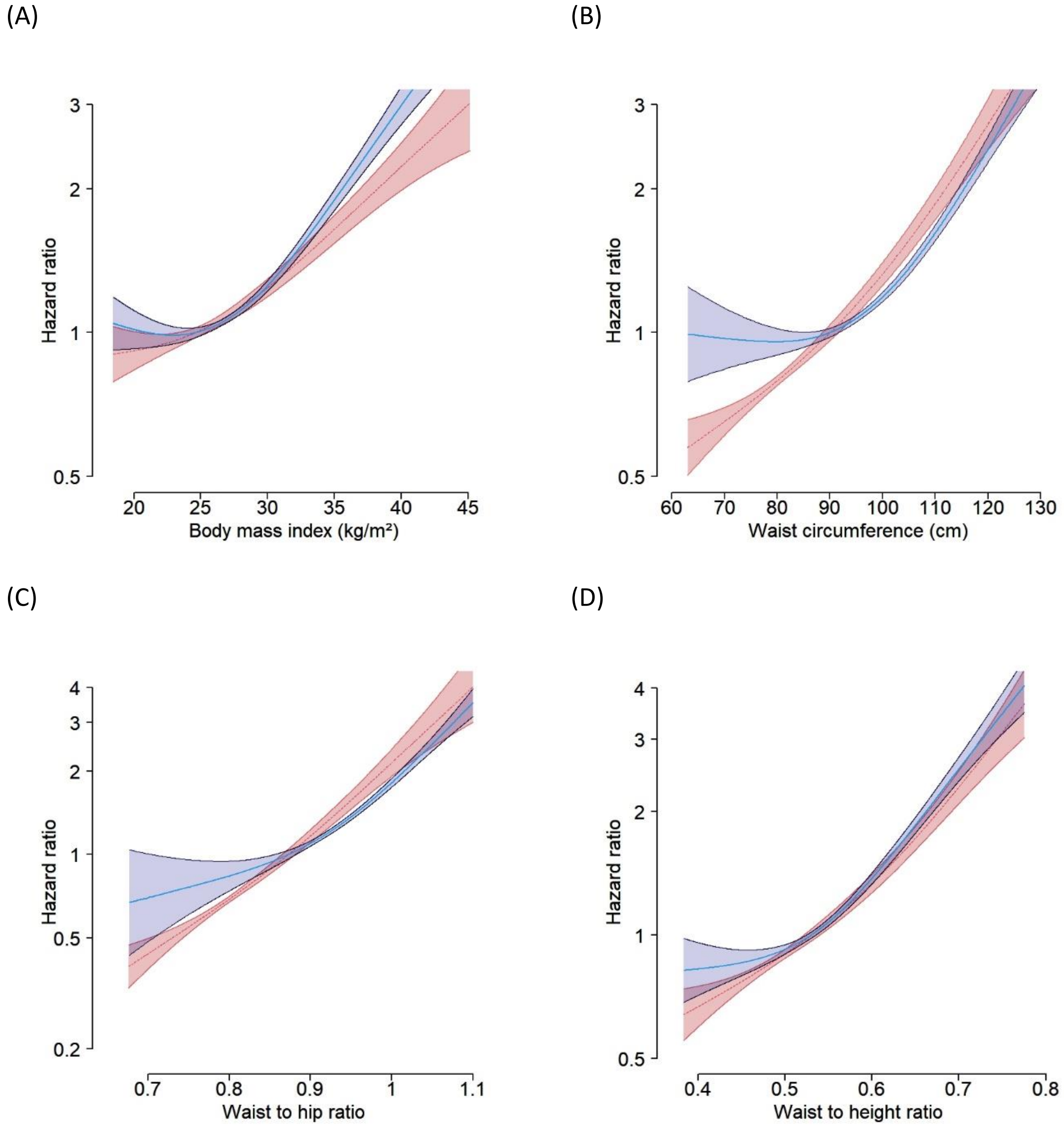

S3 Fig. Sex-specific multivariable-adjusted hazard ratios for adiposity measures with the risk of peripheral artery disease.

Modelled with penalised smoothing splines, adjusted for age, smoking, and socioeconomic status. Extreme values in the upper and lower 0.5% of the body adiposity distributions were excluded (ranges: BMI 18.5 to 45.2 kg/m^2, waist circumference 63 to 131 cm, waist-to-hip ratio 0.68 to 1.10, and waist -to-height ratio 0.38 to 0.78). Reference value for body mass index, waist circumference, waist-to-hip and waist-to-height ratios were the median values of 26.7 kg/m^2, 90 cm, 0.87, and 0.53, respectively. The pink dotted lines represent the hazard function for women, and the pink shaded areas are the 95% confidence intervals for women. The blue lines represent the hazard function for men, and the blue shaded areas are the 95% confidence intervals for men.
