# Supplementary material for: Sex differences in risk factors for incident peripheral artery disease hospitalisation or death: Cohort study of UK Biobank participants
Source: PLoS One. 2023 Oct 18;18(10):e0292083. doi: 10.1371/journal.pone.0292083 (PMC10584119; doi:10.1371/journal.pone.0292083)
Supplement: S4 Fig — (PDF) [file pone.0292083.s005.pdf]

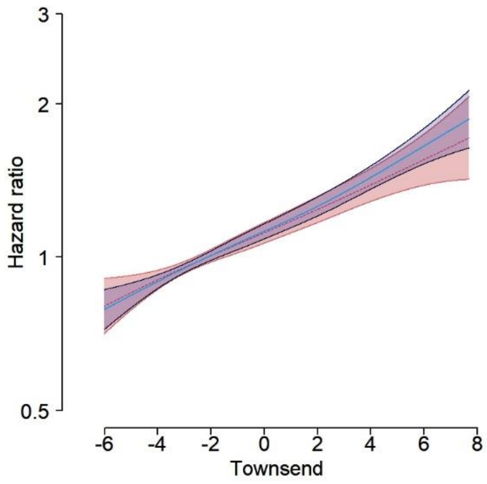

S4 Fig. Sex-specific multivariable-adjusted hazard ratios for Townsend deprivation index with the risk of peripheral artery disease.

Modelled with penalised smoothing splines, adjusted for age, diabetes, continuous measure of systolic blood pressure, body mass index, total cholesterol, estimated glomerular filtration rate calculated using cystatin C, smoking status, and lipid lowering and/or antihypertensive medications. Extreme values in the upper and lower 0.5% of the Townsend deprivation index distributions were excluded (range: -6.0 to 7.7). Reference value was the median value of -2.17. The pink dotted line represents the hazard function for women, and the pink shaded area is the 95% confidence intervals for women. The blue line represents the hazard function for men, and the blue shaded area is the 95% confidence intervals for men.
