# Supplementary material for: Sex differences in risk factors for incident peripheral artery disease hospitalisation or death: Cohort study of UK Biobank participants
Source: PLoS One. 2023 Oct 18;18(10):e0292083. doi: 10.1371/journal.pone.0292083 (PMC10584119; doi:10.1371/journal.pone.0292083)
Supplement: S5 Fig — (PDF) [file pone.0292083.s006.pdf]

(A) (B)

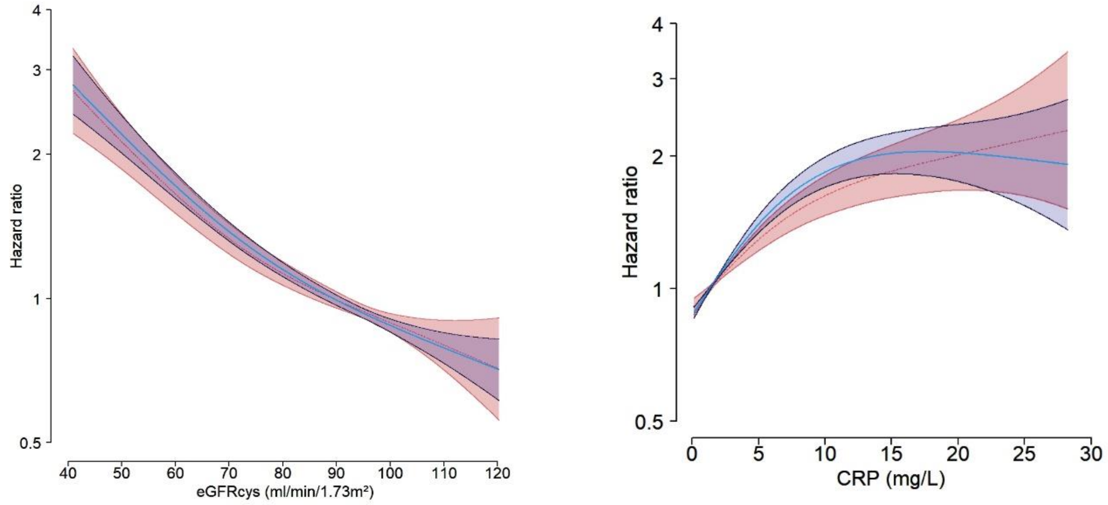

S5 Fig. Sex-specific multivariable-adjusted hazard ratios for eGFRcys and CRP with the risk of peripheral artery disease.

CRP C-reactive protein, eGFRcys estimated Glomerular Filtration Rate calculated using cystatin C. Modelled with penalised smoothing splines, adjusted for age, continuous measure of systolic blood pressure, body mass index, eGFRcys (for CRP), diabetes, total cholesterol, smoking and socioeconomic status, and lipid lowering and/or antihypertensive medications. Extreme values in the upper and lower 0.5% of the distributions were excluded (ranges: eGFRcys 40.9 to 120.3 ml/min/1.73m^2 and CRP 0.1 to 28.3 mg/L). Reference value for eGFRcys and CRP were the median values of 89.3 ml/min/1.73m^2 and 1.3 mg/L, respectively. The pink dotted lines represent the hazard function for women, and the pink shaded areas are the 95% confidence intervals for women. The blue lines represent the hazard function for men, and the blue shaded areas are the 95% confidence intervals for men.
