# Supplementary material for: Sex differences in risk factors for incident peripheral artery disease hospitalisation or death: Cohort study of UK Biobank participants
Source: PLoS One. 2023 Oct 18;18(10):e0292083. doi: 10.1371/journal.pone.0292083 (PMC10584119; doi:10.1371/journal.pone.0292083)
Supplement: S1 Table — (PDF) [file pone.0292083.s007.pdf]

S1 Table. Risk factors considered in the study and adjustments.

Risk factors	Definitions, measurements, and/or categorises	Adjusted variables[a]
Blood pressure	An average of two sitting SBPs and DBPs measured by the Omron HEM-7015IT, were used as continuous measures (per 10 mmHg higher SBP, or per 5 mmHg higher DBP, respectively). Pulse pressure (SBP minus DBP) was used as a continuous measure (per 5 mmHg higher).<br>Based on the American Heart Association 2017 guidelines [1], BP was categorised into four groups: 1) normal (SBP <120 mmHg and DBP <80 mmHg); 2) elevated (SBP 120-9 mmHg and DBP <80 mmHg); 3) stage 1 hypertension (SBP 130-9 mmHg or DBP 80-9 mmHg); and 4) stage 2 hypertension (SBP ≥140 mmHg or DBP ≥90 mmHg).	age, smoking status, diabetes, total cholesterol, BMI, Townsend deprivation index, eGFRcys, and antihypertensive and/or lipid lowering medications<br>Regarding self-reported antihypertensive and/or lipid lowering medications, in the touchscreen questionnaire, it was asked "Do you regularly take any of the following medications? (you can select more than one answer)". Available answers were "blood pressure medication", "cholesterol lowering medication", "insulin", "none of the above", "do not know", and "prefer not to answer". Based on the answers, use of lipid lowering, and antihypertensive medication was each coded as "yes" and "no".
Smoking	Smoking status was self-reported as never, former, or current smokers. Current smokers` daily consumption of cigarettes was categorised into three groups (1-9, 10-19 and ≥20 cigarettes).<br>The following two variables were not examined as risk factors but were reported as baseline characteristics: 1) smoking pack-years among current smokers: cigarettes per day multiplied by the number of years smoked, divided by 20; and 2) years since quitting among former smokers: calculated as the differences between ages at baseline and at smoking cessation.	age, Townsend deprivation index
Diabetes	Self-reported history of diabetes was collected through the touchscreen questionnaire, participants were asked "Has a doctor ever told you that you have diabetes" and confirmed in the interview. Participants were classified as type 1 diabetes if diagnosed at younger than 30 years old and using insulin, otherwise as type 2 diabetes.	age, SBP, smoking status, total cholesterol, BMI, Townsend deprivation index, eGFRcys, and antihypertensive and/or lipid lowering medications
Lipids	Blood cholesterol levels (total cholesterol, and high- and low-density lipoprotein cholesterols) were measured from blood samples using the Beckman Coulter AU5800 and used as continuous measures (per 1 mmol/L higher). Elevated total cholesterol was defined as total cholesterol ≥6.2 mmol/L. Five groups were defined based on high-density lipoprotein cholesterols levels: 30, 40, 60, 80 mg/dL, equivalent to 0.78, 1.03, 1.55, and 2.07 mmol/L [2, 3]. Participants in the first two groups were combined into one group in the Cox and Fine and Gray models, due to small sample sizes.	age, SBP, smoking status, diabetes, BMI, Townsend deprivation index, eGFRcys, and antihypertensive and/or lipid lowering medications

Risk factors	Definitions, measurements, and/or categorises	Adjusted variables[a]
Adiposity	Participants` weight (in kilograms, kg) was measured using the Tanita BC-418 MA body composition analyser (Tanita Corporation of America). Standing height (in metres, m) was measured using a Seca 202 height measure (SECA, Germany). Per 5 kg/m^2 higher BMI was examined as a risk factor. BMI (kg/m^2) was also categorised into: 1) underweight (<18); 2) healthy weight (≥18 and <25); 3) overweight (≥25 and <30); 4) obese (≥30).<br><br>Waist and hip circumferences were measured using the Wessex non-stretchable sprung tape measure. Waist circumference was used as a continuous measure (per 10 cm higher). Waist-to-hip and waist-to-height ratios (waist circumference divided by hip circumference and standing height, respectively, in same units, per 0.1 units higher) were also used.	age, smoking status, and Townsend deprivation index
Prior stroke or myocardial infarction	Self-reported history of stroke or myocardial infarction was collected through the touchscreen questionnaire and confirmed in the interview. Participants were asked "Has a doctor ever told you that you have had any of the following conditions? (you can select more than one answer)" Available answers were "heart attack", "angina", "stroke", "high blood pressure", and "none of the above". Based on the answers, prior stroke and myocardial infarction was each coded as "yes" and "no".	age, and Townsend deprivation index
Socioeconomic status	Socioeconomic status was determined using the Townsend deprivation index, a measure of area deprivation, derived from the national census data about unemployment, car ownership, household overcrowding, and owner occupation, with higher scores indicating higher levels of deprivation. The participants were then grouped into five groups based on the national cut-offs for equal fifths using the index, with the 1st group containing the least socioeconomically deprived (the lowest Townsend scores) and the 5th group containing the most deprived (the highest Townsend scores) [4].	age, SBP, smoking status, diabetes, total cholesterol, BMI, eGFRcys, and antihypertensive and/or lipid lowering medications
eGFRcys	Serum cystatin C (mg/L) was measured from blood samples by latex enhanced immunoturbidimetric analysis on a Siemens ADVIA 1800. eGFRcys was calculated using cystatin C recommended by the CKD-EPI 2021 race-free equation [5]. Per 1 ml/min/1.73m^2 eGFRcys was examined as a risk factor. eGFRcys was also categorised as normal or high (≥90 ml/min/1.73m^2) versus decreased (<90 ml/min/1.73m^2).	age, SBP, smoking status, diabetes, total cholesterol, BMI, Townsend deprivation index, and self-reported antihypertensive and/or lipid lowering medications
C-reactive protein	Serum C-reactive protein (mg/L) was measured from blood samples on the Beckman Coulter AU5800. Per 5 mg/L higher C-reactive protein was examined as a risk factor.	age, SBP, smoking status, diabetes, total cholesterol, BMI, Townsend deprivation index, eGFRcys, and self-reported

Risk factors	Definitions, measurements, and/or categorises	Adjusted variables[a]
		antihypertensive and/or lipid lowering medications
Alcohol consumption	Alcohol drinking status was self-reported as never, former, or current alcohol drinker. Frequency of alcohol consumption among current drinkers was self-reported as: special occasions only, one to three times a month, once or twice a week, three or four times a week, and daily or almost daily.	age, Townsend deprivation index, smoking status

BMI denotes body mass index, DBP diastolic blood pressure, eGFRcys estimated Glomerular Filtration Rate was calculated using cystatin C, SBP systolic blood pressure.

Reference for S1 Table

1. Whelton PK, Carey RM, Aronow WS, Casey DE, Collins KJ, Dennison Himmelfarb C, et al. 2017 ACC/AHA/AAPA/ABC/ACPM/AGS/APhA/ASH/ASPC/NMA/PCNA guideline for the prevention, detection, evaluation, and management of high blood pressure in adults: a report of the American College of Cardiology/American Heart Association task force on clinical practice guidelines. J Am Coll Cardiol. 2018;71(19):e127-e248. doi: 10.1016/j.jacc.2017.11.006.
2. Appendix A lipid conversion factors. Screening and treatment of subclinical hypothyroidism or hyperthyroidism.
3. Liu C, Dhindsa D, Almuwaqqat Z, Ko YA, Mehta A, Alkhoder AA, et al. Association between high-density lipoprotein cholesterol levels and adverse cardiovascular outcomes in high-risk populations. JAMA Cardiol. 2022;7(7):672-680. doi: 10.1001/jamacardio.2022.0912.
4. Yousaf S, Bonsall A. UK Townsend deprivation scores from 2011 census data: UK Data Service; 2017 [Available from: https://s3-eu-west-1.amazonaws.com/statistics.digitalresources.jisc.ac.uk/dkan/files/Townsend_Deprivation_Scores/UK%20Townsend%20Deprivation%20Scores%20from%202011%20census%20data.pdf [accessed 21 April 2023].
5. Miller WG, Kaufman HW, Levey AS, Straseski JA, Wilhelms KW, Yu HYE, et al. National Kidney Foundation Laboratory Engagement Working Group recommendations for implementing the CKD-EPI 2021 race-free equations for estimated glomerular filtration rate: practical guidance for clinical laboratories. Clin Chem. 2022;68(4):511-520. doi: 10.1093/clinchem/hvab278.
