# Supplementary material for: Sex differences in risk factors for incident peripheral artery disease hospitalisation or death: Cohort study of UK Biobank participants
Source: PLoS One. 2023 Oct 18;18(10):e0292083. doi: 10.1371/journal.pone.0292083 (PMC10584119; doi:10.1371/journal.pone.0292083)
Supplement: S2 Table — (PDF) [file pone.0292083.s008.pdf]

S2 Table. Diagnostic and procedure codes used in ascertaining peripheral artery disease.

ICD-9	ICD 10	OPCS	
		OPCS	Rule out ICD-9 or -10 codes for aneurysms before or at the time of the OPCS in a sensitivity analysis
440.2 (440.20-4, 440.29)	I70.0 (I70.00-1)	L22.2	442.0, I72.1
440.3 (440.30-2)	I70.2 (I70.20-1)	L26.1-2	
440.4	I70.8 (I70.80-1)	L37	442.0, I72.1
440.8	I70.9 (I70.90-1)	L37.1	442.0, I72.1
440.9	I73.9	L37.3-4	
443.9	I79.2	L37.8-9	442.0, I72.1
250.7 (250.70-3)	E11.5	L38.1	442.0, I72.1
		L38.8-9	442.0, I72.1
		L39.1	
		L39.5	442.0, I72.1
		L39.8-9	442.0, I72.1
		L50 (L50.1-6, L50.8-9)	442.2, I72.3
		L51 (L51.1-6, L51.8-9)	442.2, I72.3
		L52	442.2, I72.3
		L52.1-2	
		L52.8-9	442.2, I72.3
		L53.8-9	442.2, I72.3
		L54.1	
		L54.4	442.2, I72.3
		L54.8-9	442.2, I72.3
		L58 (L58.1-9)	442.3, I72.4
		L59 (L59.1-9)	442.3, I72.4
		L60	442.3, I72.4
		L60.1-4	
		L60.8-9	442.3, I72.4
		L62.1	442.3, I72.4
		L62.8-9	442.3, I72.4
		L63.1	
		L63.5	442.3, I72.4
		L63.8-9	442.3, I72.4
		L65.2-3	442.3, I72.4
		L65.8-9	442.0, 442.2, 442.3, I72.1, I72.3, I72.4
		L66	442.0, 442.2, 442.3, I72.1, I72.3, I72.4
		L66.1	
		L66.2	442.0, 442.2, 442.3, I72.1, I72.3, I72.4
		L66.5	
		L66.7	442.0, 442.2, 442.3, I72.1, I72.3, I72.4
		L66.8-9	442.0, 442.2, 442.3, I72.1, I72.3, I72.4
		L68	442.0, 442.2, 442.3, I72.1, I72.3, I72.4
		L68.1-2	
		L68.3-4	442.0, 442.2, 442.3, I72.1, I72.3, I72.4
		L68.8-9	442.0, 442.2, 442.3, I72.1, I72.3, I72.4
		L71.1	
		L71.5-7	
		L71.8-9	442.0, 442.2, 442.3, I72.1, I72.3, I72.4

		L76	442.0, 442.2, 442.3, I72.1, I72.3, I72.4
		L76.1-9	
		L89 (L89.1-6, L89.8-9)	

ICD-9 and -10 denotes International Classification of Disease ninth and tenth revision, OPCS Office of Population Censuses and Surveys Classification of Surgical Operations and Procedures.
