# Supplementary material for: Sex differences in risk factors for incident peripheral artery disease hospitalisation or death: Cohort study of UK Biobank participants
Source: PLoS One. 2023 Oct 18;18(10):e0292083. doi: 10.1371/journal.pone.0292083 (PMC10584119; doi:10.1371/journal.pone.0292083)
Supplement: S3 Table — (PDF) [file pone.0292083.s009.pdf]

S3 Table. Baseline characteristics of the UK Biobank participants excluded from the current analyses due to a previous hospital admission with peripheral artery disease.

Characteristics	Prior hospitalisation with PAD at baseline (n=2206)		
	Women (n=624)	Men (n=1582)	Women & Men (n=2206)
Age (years, mean (SD))	60.7 (6.7)	61.9 (6.2)	61.6 (6.4)
Ethnicity			
White	586 (93.9)	1504 (95.1)	2090 (94.7)
Other	38 (6.1)	78 (4.9)	116 (5.3)
Systolic blood pressure (mmHg, mean (SD))	139.7 (20.5)	143.7 (20.3)	142.6 (20.4)
Diastolic blood pressure (mmHg, mean (SD))	78.2 (10.8)	79.4 (10.7)	79.1 (10.8)
Pulse pressure (mmHg, mean (SD))	61.5 (18.0)	64.3 (16.7)	63.5 (17.1)
AHA hypertension categories			
Normal	85 (13.7)	164 (10.4)	249 (11.4)
Elevated	93 (15.0)	166 (10.6)	259 (11.8)
Stage 1 hypertension	139 (22.4)	338 (21.5)	477 (21.8)
Stage 2 hypertension	304 (49.0)	903 (57.5)	1207 (55.1)
Smoking status			
Never	205 (33.3)	256 (16.3)	461 (21.1)
Former	257 (41.7)	894 (57.0)	1151 (52.7)
Current	154 (25.0)	418 (26.7)	572 (26.2)
Pack-years among current smokers, mean (SD)	36.3 (17.1)	43.7 (22.4)	41.5 (21.2)
Year since quitting among former smokers, mean (SD)	12.8 (10.6)	13.1 (10.5)	13 (10.5)
Smoking intensity (cigarettes per day)[a]			
≤9	11 (7.1)	30 (7.2)	41 (7.2)
10-19	63 (40.9)	93 (22.2)	156 (27.3)
≥20	53 (34.4)	176 (42.1)	229 (40)
Not reported	27 (17.5)	119 (28.5)	146 (25.5)
Type 1 diabetes	17 (2.8)	34 (2.2)	51 (2.3)
Type 2 diabetes[b]	117 (18.9)	401 (25.7)	518 (23.8)
Cholesterol (mmol/L)			
Total cholesterol (mean (SD))	5.2 (1.2)	4.6 (1.1)	4.7 (1.2)
HDL-C (mean (SD))	1.4 (0.4)	1.2 (0.3)	1.2 (0.4)
LDL-C (mean (SD))	3.1 (0.9)	2.8 (0.8)	2.9 (0.9)
Elevated total cholesterol (≥6.2 mmol/L)	94 (16.7)	121 (8.4)	(10.7)
HDL-C categories			
≤0.78	10 (2.0)	90 (6.8)	100 (5.5)
>0.78 and ≤1.03	58 (11.4)	422 (32.0)	480 (26.3)
>1.03 and ≤1.55	275 (53.9)	653 (49.6)	928 (50.8)
>1.55 and ≤2.07	137 (26.9)	121 (9.2)	258 (14.1)
>2.07	30 (5.9)	31 (2.4)	61 (3.3)
BMI (kg/m^2, mean (SD))	28.9 (6.0)	29.0 (4.8)	29.0 (5.1)
BMI (kg/m^2) categories			
Underweight (<18.5)	6 (1.0)	10 (0.7)	16 (0.7)
Healthy weight (18.5-24.9)	169 (27.7)	280 (18.3)	449 (21.0)
Overweight (25-29.9)	199 (32.6)	664 (43.3)	863 (40.3)
Obese (≥30)	237 (38.8)	578 (37.7)	815 (38.0)
Waist circumference (cm, mean (SD))	91.7 (14.2)	101.6 (12.5)	98.8 (13.7)

Waist-to-hip ratio (mean (SD))	0.87 (0.07)	0.98 (0.07)	0.95 (0.09)
Waist-to-height ratio (mean (SD))	0.57 (0.09)	0.59 (0.07)	0.58 (0.08)
History of stroke	62 (9.9)	198 (12.5)	260 (11.8)
History of myocardial infarction	71 (11.4)	425 (26.9)	496 (22.5)
Socioeconomic status (fifths)[c]			
1st (least deprived)	142 (22.8)	401 (25.4)	543 (24.6)
2nd	107 (17.2)	272 (17.2)	379 (17.2)
3rd	94 (15.1)	182 (11.5)	276 (12.5)
4th	108 (17.3)	285 (18.0)	393 (17.8)
5th (most deprived)	172 (27.6)	440 (27.8)	612 (27.8)
eGFRcys (ml/min/1.73m^2, mean (SD))	71.3 (58.1, 86.2)	74.5 (60.1, 87.2)	73.6 (59.3, 87)
eGFRcys (ml/min/1.73m^2) categories			
G1 normal or high (≥90)	113 (20.0)	304 (21.0)	417 (20.7)
G2 Mildly decreased (60-89)	290 (51.3)	786 (54.2)	1076 (53.4)
G3a Mildly to moderately decreased (45-59)	100 (17.7)	214 (14.8)	314 (15.6)
G3b Moderately to severely decreased (30-44)	44 (7.8)	99 (6.8)	143 (7.1)
G4 Severely decreased (15-29)	13 (2.3)	41 (2.8)	54 (2.7)
G5 kidney failure (<15)	5 (0.9)	6 (0.4)	11 (0.5)
eGFRcys (ml/min/1.73m^2) categories			
Normal or high (≥90)	113 (20.0)	304 (21.0)	417 (20.7)
Decreased (<90)	452 (80.0)	1146 (79.0)	1598 (79.3)
C-reactive protein (mg/L, median (Q1, Q3))	2.6 (1.4, 5.7)	2.3 (1.1, 4.9)	2.4 (1.1, 5.2)
Alcohol drinker status			
Never	68 (11.0)	39 (2.5)	107 (4.9)
Previous	68 (11.0)	131 (8.3)	199 (9.1)
Current	485 (78.1)	1402 (89.2)	1887 (86.0)
Frequency of alcohol consumption[d]			
Special occasions only	144 (29.7)	177 (12.6)	321 (17.0)
One to three times a month	82 (16.9)	145 (10.3)	227 (12.0)
Once or twice a week	120 (24.7)	375 (26.7)	495 (26.2)
Three or four times a week	67 (13.8)	312 (22.3)	379 (20.1)
Daily or almost daily	72 (14.8)	393 (28.0)	465 (24.6)
Medication use			
Antihypertensive medication	347 (55.6)	993 (62.8)	1340 (60.7)
Lipid-lowering medication	391 (62.7)	1224 (77.4)	1615 (73.2)

AHA denotes American Heart Association, BMI body mass index, eGFRcys estimated Glomerular Filtration Rate calculated using cystatin C, HDL-C high-density lipoprotein cholesterol, LDL-C low-density lipoprotein cholesterol, PAD peripheral artery disease, Q1 the first quartile, Q3 the third quartile, SD standard deviation.

Numbers are numbers of participants (percentages within women, men, and overall study sample), unless when specified as mean and SD or median (Q1, Q3).

[a]As smoking intensity was only collected from current smokers, the denominators are the number of women and/or men who reported themselves as current smokers.

[b]Defined as diagnosis before the age of 30 years old and receiving insulin treatment.

[c]Socioeconomic status was determined using the Townsend Deprivation Index and grouped into five groups based on the cut-offs for the UK national equal fifths, with the 1st group containing the least socially deprived and the 5th group the most deprived.

[d]As frequency of alcohol consumption was only collected from current alcohol drinkers, the denominators are the number of women and/or men who reported themselves as current alcohol drinkers.
