# Supplementary material for: Sex differences in risk factors for incident peripheral artery disease hospitalisation or death: Cohort study of UK Biobank participants
Source: PLoS One. 2023 Oct 18;18(10):e0292083. doi: 10.1371/journal.pone.0292083 (PMC10584119; doi:10.1371/journal.pone.0292083)
Supplement: S4 Table — (PDF) [file pone.0292083.s010.pdf]

S4 Table. Baseline characteristics of the UK Biobank participants included in the current analyses by PAD status.

Characteristics	No PAD (n = 492547)			Incident PAD (n = 7660)			Overall (n = 500207)
	Women	Men	Women&Men	Women	Men	Women&Men	
Age (years, mean (SD))	56.3 (8.0)	56.6 (8.2)	56.4 (8.1)	61.3 (6.7)	61.6 (6.4)	61.5 (6.5)	56.5 (8.1)
Ethnicity							
White	254040 (94.1)	208663 (93.8)	462703 (93.9)	2522 (94.9)	4732 (94.6)	7254 (94.7)	469957 (94.0)
Other	16006 (5.9)	13838 (6.2)	29844 (6.1)	136 (5.1)	270 (5.4)	406 (5.3)	30250 (6.0)
Systolic blood pressure (mmHg, mean (SD))	135.2 (19.2)	140.8 (17.4)	137.7 (18.6)	142.9 (21.0)	145.4 (19.5)	144.5 (20.1)	137.8 (18.7)
Diastolic blood pressure (mmHg, mean (SD))	80.7 (10.0)	84.2 (10.0)	82.3 (10.1)	80.5 (10.6)	82.7 (10.8)	81.9 (10.8)	82.3 (10.2)
Pulse pressure (mmHg, mean (SD))	54.5 (14.2)	56.6 (12.6)	55.4 (13.6)	62.4 (17.2)	62.7 (15.7)	62.6 (16.2)	55.6 (13.7)
AHA hypertension categories							
Normal	54921 (20.4)	19550 (8.8)	74471 (15.2)	312 (11.8)	345 (6.9)	657 (8.6)	75128 (15.1)
Elevated	35121 (13.0)	25320 (11.4)	60441 (12.3)	269 (10.2)	493 (9.9)	762 (10.0)	61203 (12.3)
Stage 1 hypertension	71401 (26.5)	61282 (27.6)	132683 (27.0)	582 (22.0)	1104 (22.2)	1686 (22.1)	134369 (26.9)
Stage 2 hypertension	107853 (40.0)	115824 (52.2)	223677 (45.5)	1477 (55.9)	3042 (61.0)	4519 (59.3)	228196 (45.7)
Smoking status							
Never	160834 (59.9)	109987 (49.7)	270821 (55.3)	985 (37.5)	1211 (24.4)	2196 (28.9)	273017 (54.9)
Former	84227 (31.4)	84287 (38.1)	168514 (34.4)	947 (36.0)	2413 (48.7)	3360 (44.3)	171874 (34.6)
Current	23509 (8.8)	26851 (12.1)	50360 (10.3)	698 (26.5)	1332 (26.9)	2030 (26.8)	52390 (10.5)
Smoking intensity (cigarettes per day)[a]							
≤9	4305 (18.3)	2693 (10.0)	6998 (13.9)	87 (12.5)	87 (6.5)	174 (8.6)	7172 (13.7)
10-19	8104 (34.5)	6518 (24.3)	14622 (29.0)	274 (39.3)	383 (28.8)	657 (32.4)	15279 (29.2)
≥20	5295 (22.5)	7210 (26.9)	12505 (24.8)	241 (34.5)	528 (39.6)	769 (37.9)	13274 (25.3)
Not reported	5805 (24.7)	10430 (38.8)	16235 (32.2)	96 (13.8)	334 (25.1)	430 (21.2)	16665 (31.8)
Pack-years among current smokers, mean (SD)	25.6 (15.6)	31.6 (20. 1)	28.5 (18.2)	34.1 (17.3)	40.7 (21.1)	38.2 (20.0)	28.9 (18.3)
Year since quitting among former smokers, mean (SD)	18.1 (11.6)	19.3 (12.1)	18.7 (11.9)	14.7 (11.5)	16.4 (12.1)	15.9 (12.0)	18.7 (11.9)
Type 1 diabetes	408 (0.2)	498 (0.2)	906 (0.2)	30 (1.1)	52 (1.0)	82 (1.1)	988 (0.2)
Type 2 diabetes[b]	9443 (3.5)	13805 (6.2)	23248 (4.7)	382 (14.5)	1208 (24.4)	1590 (20.9)	24838 (5.0)
Cholesterol (mmol/L, mean (SD))							
Total cholesterol	5.9 (1.1)	5.5 (1.1)	5.7 (1.1)	5.7 (1.3)	5.0 (1.2)	5.3 (1.3)	5.7 (1.1)

HDL-C	1.6 (0.4)	1.3 (0.3)	1.5 (0.4)	1.5 (0.4)	1.2 (0.3)	1.3 (0.4)	1.4 (0.4)
LDL-C	3.6 (0.9)	3.5 (0.9)	3.6 (0.9)	3.5 (1.0)	3.1 (0.9)	3.3 (1.0)	3.6 (0.9)
Elevated total cholesterol (≥6.2 mmol/L)	92278 (36.7)	53669 (25.7)	145947 (31.7)	825 (34.0)	791 (17.1)	1616 (22.9)	147563 (31.6)
HDL-C categories							
≤0.78	491 (0.2)	3484 (1.8)	3975 (0.9)	17 (0.8)	220 (5.1)	237 (3.7)	4212 (1.0)
>0.78 and ≤1.03	9343 (4.1)	37382 (19.4)	46725 (11.1)	228 (10.4)	1239 (29.0)	1467 (22.6)	48192 (11.3)
>1.03 and ≤1.55	103773 (45.4)	118914 (61.7)	222687 (52.8)	1147 (52.1)	2270 (53.0)	3417 (52.7)	226104 (52.8)
>1.55 and ≤2.07	90095 (39.4)	29448 (15.3)	119543 (28.4)	651 (29.6)	442 (10.3)	1093 (16.9)	120636 (28.2)
>2.07	24927 (10.9)	3632 (1.9)	28559 (6.8)	158 (7.2)	108 (2.5)	266 (4.1)	28825 (6.7)
BMI (kg/m^2, mean (SD))	27.1 (5.2)	27.8 (4.2)	27.4 (4.8)	28.7 (6.3)	29.1 (5.1)	28.9 (5.6)	27.4 (4.8)
BMI (kg/m^2) categories							
Underweight (<18.5)	2037 (0.8)	516 (0.2)	2553 (0.5)	36 (1.4)	21 (0.4)	57 (0.8)	2610 (0.5)
Healthy weight (18.5-24.9)	104728 (39.0)	55437 (25.1)	160165 (32.7)	743 (28.4)	1017 (20.6)	1760 (23.3)	161925 (32.6)
Overweight (25-29.9)	98709 (36.7)	109522 (49.6)	208231 (42.5)	945 (36.2)	2040 (41.4)	2985 (39.6)	211216 (42.5)
Obese (≥30)	63172 (23.5)	55500 (25.1)	118672 (24.2)	889 (34.0)	1854 (37.6)	2743 (36.4)	121415 (24.4)
Waist circumference (cm, mean (SD))	84.7 (12.5)	96.8 (11.3)	90.2 (13.4)	90.7 (15.0)	101.4 (13.4)	97.7 (14.9)	90.3 (13.5)
Waist-to-hip ratio (mean (SD))	0.82 (0.07)	0.93 (0.06)	0.87 (0.09)	0.86 (0.08)	0.93 (0.07)	0.93 (0.09)	0.9 (0.10)
Waist-to-height ratio (mean (SD))	0.52 (0.08)	0.55 (0.07)	0.54 (0.07)	0.56 (0.09)	0.58 (0.08)	0.58 (0.08)	0.5 (0.10)
History of stroke	2957 (1.1)	3987 (1.8)	6944 (1.4)	133 (5.0)	330 (6.6)	463 (6.0)	7407 (1.5)
History of myocardial infarction	2078 (0.8)	8143 (3.7)	10221 (2.1)	151 (5.7)	737 (14.7)	888 (11.6)	11109 (2.2)
Socioeconomic status[c]							
1st (least deprived)	100083 (37.1)	83058 (37.4)	183141 (37.2)	755 (28.4)	1411 (28.2)	2166 (28.3)	185307 (37.1)
2nd	56008 (20.8)	44977 (20.2)	100985 (20.5)	468 (17.6)	925 (18.5)	1393 (18.2)	102378 (20.5)
3rd	40727 (15.1)	32430 (14.6)	73157 (14.9)	418 (15.7)	721 (14.4)	1139 (14.9)	74296 (14.9)
4th	36370 (13.5)	29451 (13.3)	65821 (13.4)	422 (15.9)	716 (14.3)	1138 (14.9)	66959 (13.4)
5th (most deprived)	36536 (13.5)	32295 (14.5)	68831 (14.0)	591 (22.3)	1225 (24.5)	1816 (23.7)	70647 (14.1)
eGFRcys (ml/min/1.73m^2, mean (SD))	88.8 (15.9)	88.2 (16.2)	88.5 (16.0)	75.8 (19.0)	76.4 (19.2)	76.2 (19.1)	88.3 (16.2)
eGFRcys (ml/min/1.73m^2) categories							
G1 normal or high (≥90)	127703 (50.8)	97107 (46.5)	224810 (48.8)	607 (25.0)	1096 (23.6)	1703 (24.1)	226513 (48.5)
G2 Mildly decreased (60-89)	112881 (44.9)	102614 (49.1)	215495 (46.8)	1352 (55.8)	2732 (58.9)	4084 (57.8)	219579 (47.0)
G3a Mildly to moderately decreased (45-59)	9296 (3.7)	7397 (3.5)	16693 (3.6)	312 (12.9)	539 (11.6)	851 (12.1)	17544 (3.8)
G3b Moderately to severely decreased (30-44)	1421 (0.6)	1362 (0.7)	2783 (0.6)	114 (4.7)	183 (3.9)	297 (4.2)	3080 (0.7)

G4 Severely decreased (15-29)	238 (0.1)	292 (0.1)	530 (0.1)	28 (1.2)	71 (1.5)	99 (1.4)	629 (0.1)
G5 kidney failure (<15)	35 (0)	53 (0)	88 (0)	12 (0.5)	16 (0.3)	28 (0.4)	116 (0)
eGFRcys (ml/min/1.73m^2) categories							
Normal or high (≥90)	127703 (50.8)	97107 (46.5)	224810 (48.8)	607 (25.0)	1096 (23.6)	1703 (24.1)	226513 (48.5)
Decreased (<90)	123871 (49.2)	111718 (53.5)	235589 (51.2)	1818 (75.0)	3541 (76.4)	5359 (75.9)	240948 (51.5)
C-reactive protein (mg/L, median (Q1, Q3))	1.37 (0.65, 2.95)	1.26 (0.66, 2.50)	1.32 (0.65, 2.73)	2.39 (1.08, 4.99)	2.06 (1.04, 4.20)	2.17 (1.04, 4.45)	1.33 (0.66, 2.76)
Alcohol drinker status							
Never	15681 (5.8)	6215 (2.8)	21896 (4.5)	227 (8.6)	151 (3.0)	378 (5.0)	22274 (4.5)
Previous	9700 (3.6)	7673 (3.5)	17373 (3.5)	209 (7.9)	313 (6.3)	522 (6.9)	17895 (3.6)
Current	243857 (90.6)	207829 (93.7)	451686 (92.0)	2205 (83.5)	4508 (90.7)	6713 (88.2)	458399 (91.9)
Frequency of alcohol consumption[d]							
Special occasions only	40421 (16.6)	16162 (7.8)	56583 (12.5)	580 (26.3)	513 (11.4)	1093 (16.3)	57676 (12.6)
One to three times a month	35080 (14.4)	19768 (9.5)	54848 (12.1)	333 (15.1)	433 (9.6)	766 (11.4)	55614 (12.1)
Once or twice a week	69513 (28.5)	57545 (27.7)	127058 (28.1)	531 (24.1)	1186 (26.3)	1717 (25.6)	128775 (28.1)
Three or four times a week	55461 (22.7)	58231 (28.0)	113692 (25.2)	359 (16.3)	994 (22.0)	1353 (20.2)	115045 (25.1)
Daily or almost daily	43382 (17.8)	56123 (27.0)	99505 (22.0)	402 (18.2)	1382 (30.7)	1784 (26.6)	101289 (22.1)
Medication use							
Antihypertensive medication	46458 (17.2)	52428 (41.7)	98886 (20.1)	1108 (23.6)	2653 (53.0)	3761 (49.1)	102647 (20.5)
Lipid-lowering medication	33237 (12.3)	48450 (21.8)	81687 (16.6)	980 (36.9)	2596 (51.9)	3576 (46.7)	85263 (17.0)

AHA denotes American Heart Association, BMI body mass index, eGFRcys estimated Glomerular Filtration Rate calculated using cystatin C, HDL-C high-density lipoprotein cholesterol, LDL-C low-density lipoprotein cholesterol, PAD peripheral artery disease, Q1 the first quartile, Q3 the third quartile, SD standard deviation.

Numbers are numbers of participants (percentages within women, men, and overall study sample), unless when specified as mean and SD. Denominators for the percentages are the total number of women, men, or both in the current study sample with and without peripheral artery disease.

[a]As smoking intensity was only collected from current smokers, the denominators are the number of women and men who reported themselves as current smokers.

[b]Defined as diagnosis before the age of 30 years old and receiving insulin treatment.

[c]Socioeconomic status was determined using the Townsend Deprivation Index and grouped into five groups based on the cut-offs for the UK national equal fifths, with the 1st group containing the least socially deprived and the 5th group the most deprived.

[d]As frequency of alcohol consumption was only collected from current alcohol drinkers, the denominators are the number of women and men who reported themselves as current alcohol drinkers.
