# Supplementary material for: Sex differences in risk factors for incident peripheral artery disease hospitalisation or death: Cohort study of UK Biobank participants
Source: PLoS One. 2023 Oct 18;18(10):e0292083. doi: 10.1371/journal.pone.0292083 (PMC10584119; doi:10.1371/journal.pone.0292083)
Supplement: S5 Table — (PDF) [file pone.0292083.s011.pdf]

S5 Table. Numbers and percentages of missing values for each variable.

Variable	Missing values	
	Number of participants	Percentage (total n=500,207, unless specified)
Age	0	0
Ethnicity	0	0
Systolic blood pressure	1311	0.3
Diastolic blood pressure	1309	0.3
Pulse pressure	1311	0.3
AHA hypertension categories	1311	0.3
Smoking status	2926	0.6
Type 1 or 2 diabetes	2587	0.5
Cholesterol (mmol/L)		
Total cholesterol	32713	6.5
HDL-C	72238	14.4
LDL-C	33590	6.7
BMI	3041	0.6
Waist circumference	2131	0.4
Waist-to-hip ratio	2230	0.4
Waist-to-height ratio	2626	0.5
History of stroke	0	0
History of myocardial infarction	0	0
Socioeconomic status[a]	620	0.1
eGFRcys	32746	6.5
C-reactive protein	33720	6.7
Medication use	0	0
Alcohol drinker status	1639	0.3
Frequency of alcohol consumption[b]	0	0

AHA denotes American Heart Association, BMI body mass index, eGFRcys estimated Glomerular Filtration Rate calculated using cystatin C, HDL-C high-density lipoprotein cholesterol, LDL-C low-density lipoprotein cholesterol.

[a]Socioeconomic status was determined using the Townsend Deprivation Index.

[b]As frequency of alcohol consumption was only collected from current alcohol drinkers, the denominator is the number of women and men who reported themselves as current alcohol drinkers.
