# Supplementary material for: Sex differences in risk factors for incident peripheral artery disease hospitalisation or death: Cohort study of UK Biobank participants
Source: PLoS One. 2023 Oct 18;18(10):e0292083. doi: 10.1371/journal.pone.0292083 (PMC10584119; doi:10.1371/journal.pone.0292083)
Supplement: S6 Table — (PDF) [file pone.0292083.s012.pdf]

S6 Table. Age-adjusted sex-specific hazard ratios and women-to-men ratio of hazard ratios for risk factors.

Risk factors	Age-adjusted		
	HR (95% CI)		Women-to-men ratio of HRs (95% CI)
	Women	Men	
Systolic blood pressure, per 10 mmHg	1.09 (1.07, 1.11)	1.06 (1.04, 1.07)	1.03 (1.01, 1.06)
Diastolic blood pressure, per 5 mmHg	0.97 (0.95, 0.99)	0.93 (0.92, 0.95)	1.04 (1.02, 1.07)
Pulse pressure, per 5 mmHg	1.09 (1.08, 1.11)	1.09 (1.08, 1.10)	1.00 (0.99, 1.02)
AHA hypertension categories			
Normal	Reference	Reference	Reference
Elevated	0.99 (0.84, 1.16)	1.00 (0.87, 1.15)	0.99 (0.80, 1.22)
Stage 1 hypertension	1.05 (0.92, 1.21)	0.91 (0.80, 1.02)	1.16 (0.97, 1.40)
Stage 2 hypertension	1.38 (1.21, 1.56)	1.05 (0.94, 1.18)	1.31 (1.10, 1.55)
Smoking status			
Never	Reference	Reference	Reference
Former	1.67 (1.53, 1.82)	2.06 (1.92, 2.21)	0.81 (0.72, 0.91)
Current	5.93 (5.38, 6.54)	5.07 (4.69, 5.48)	1.17 (1.03, 1.32)
Former versus current smokers	0.28 (0.25, 0.31)	0.40 (0.38, 0.43)	0.69 (0.61, 0.78)
Current versus never or former smokers	4.78 (4.38, 5.21)	3.36 (3.16, 3.58)	1.42 (1.27, 1.58)
By smoking intensity[a]			
Never	Reference	Reference	Reference
≤9 cigarettes per day	3.92 (3.15, 4.88)	3.53 (2.84, 4.38)	1.11 (0.82, 1.51)
10-19 cigarettes per day	6.55 (5.72, 7.49)	6.24 (5.56, 7.00)	1.05 (0.88, 1.25)
≥20 cigarettes per day	9.26 (8.04, 10.67)	8.13 (7.34, 9.02)	1.14 (0.96, 1.36)
No diabetes	Reference	Reference	Reference
Type 1 diabetes	12.33 (8.60, 17.69)	7.18 (5.46, 9.44)	1.72 (1.09, 2.70)
Type 2 diabetes[b]	3.92 (3.52, 4.37)	3.97 (3.72, 4.24)	0.99 (0.87, 1.12)
Cholesterol, per 1 mmol/L			
Total cholesterol	0.79 (0.76, 0.81)	0.75 (0.73, 0.77)	1.05 (1.01, 1.10)
HDL-C	0.34 (0.30, 0.38)	0.38 (0.34, 0.42)	0.89 (0.75, 1.05)
LDL-C	0.78 (0.75, 0.82)	0.69 (0.66, 0.71)	1.14 (1.07, 1.21)
Total cholesterol			
Normal (<6.2 mmol/L)	Reference	Reference	Reference
Elevated (≥6.2 mmol/L)	0.71 (0.65, 0.77)	0.66 (0.61, 0.71)	1.08 (0.97, 1.21)
HDL-C categories			
≤1.03	2.46 (2.14, 2.82)	1.91 (1.78, 2.04)	1.29 (1.11, 1.50)
>1.03 and ≤1.55	Reference	Reference	Reference
>1.55 and ≤2.07	0.64 (0.58, 0.70)	0.75 (0.68, 0.83)	0.85 (0.74, 0.98)
>2.07	0.53 (0.45, 0.63)	1.45 (1.20, 1.76)	0.37 (0.28, 0.47)
Body mass index, per 5 kg/m^2	1.29 (1.25, 1.34)	1.38 (1.34, 1.42)	0.94 (0.90, 0.98)
Body mass index (kg/m^2) categories			
Underweight (<18.5)	2.56 (1.84, 3.58)	2.80 (1.82, 4.31)	0.92 (0.53, 1.58)
Healthy weight (18.5-24.9)	Reference	Reference	Reference
Overweight (25-29.9)	1.18 (1.07, 1.30)	0.96 (0.89, 1.04)	1.22 (1.08, 1.38)
Obese (30 and above)	1.80 (1.63, 1.99)	1.78 (1.65, 1.92)	1.01 (0.89, 1.14)
Waist circumference, per 10 cm	1.36 (1.32, 1.40)	1.34 (1.31, 1.37)	1.01 (0.98, 1.05)
Waist-to-hip ratio, per 0.1	1.44 (1.41, 1.47)	1.72 (1.67, 1.77)	0.84 (0.81, 0.87)
Waist-to-height ratio, per 0.1	1.64 (1.57, 1.71)	1.71 (1.65, 1.78)	0.96 (0.90, 1.01)
History of stroke	3.66 (3.07, 4.36)	2.93 (2.62, 3.28)	1.25 (1.02, 1.54)

Risk factors	Age-adjusted		
	HR (95% CI)		Women-to-men ratio of HRs (95% CI)
	Women	Men	
History of myocardial infarction	5.39 (4.57, 6.36)	3.38 (3.12, 3.66)	1.59 (1.33, 1.92)
Socioeconomic status[c]			
1st (least deprived)	Reference	Reference	Reference
2nd	1.13 (1.01, 1.27)	1.25 (1.15, 1.35)	0.91 (0.79, 1.04)
3rd	1.46 (1.30, 1.65)	1.45 (1.32, 1.58)	1.01 (0.87, 1.17)
4th	1.72 (1.52, 1.93)	1.67 (1.52, 1.82)	1.03 (0.89, 1.20)
5th (most deprived)	2.51 (2.26, 2.80)	2.79 (2.58, 3.01)	0.90 (0.79, 1.03)
eGFRcys, per 10 ml/min/1.73m^2	0.70 (0.68, 0.72)	0.73 (0.71, 0.74)	0.96 (0.93, 0.99)
eGFRcys (ml/min/1.73m^2) categories			
Normal or high (≥90)	Reference	Reference	Reference
Decreased (<90)	1.90 (1.72, 2.09)	1.82 (1.70, 1.96)	1.04 (0.92, 1.17)
C-reactive protein, per 1 mg/L	1.23 (1.21, 1.26)	1.20 (1.18, 1.22)	1.03 (1.00, 1.05)
Alcohol drinker status			
Never	Reference	Reference	Reference
Previous	1.57 (1.30, 1.89)	1.65 (1.36, 2.01)	0.95 (0.72, 1.24)
Current	0.69 (0.60, 0.79)	0.84 (0.71, 0.99)	0.83 (0.67, 1.02)
Frequency of alcohol consumption[d]			
Never	Reference	Reference	Reference
Special occasions only	1.04 (0.89, 1.21)	1.24 (1.03, 1.48)	0.84 (0.66, 1.06)
One to three times a month	0.76 (0.65, 0.90)	0.94 (0.78, 1.13)	0.81 (0.63, 1.04)
Once or twice a week	0.60 (0.52, 0.71)	0.84 (0.71, 1.00)	0.72 (0.57, 0.91)
Three or four times a week	0.52 (0.44, 0.61)	0.67 (0.56, 0.79)	0.77 (0.61, 0.98)
Daily or almost daily	0.66 (0.56, 0.77)	0.87 (0.73, 1.03)	0.76 (0.60, 0.95)

AHA denotes American Heart Association, CI confidence interval, eGFRcys estimated Glomerular Filtration Rate calculated using cystatin C, HDL high-density lipoprotein, LDL low-density lipoprotein.

[a]Smoking intensity was only collected from current smokers.

[b]Defined as diagnosis before the age of 30 years old and receiving insulin treatment.

[c] Socioeconomic status was determined using the Townsend Deprivation Index and grouped into five groups based on the cut-offs for the UK national equal fifths, with the 1st group containing the least socially deprived and the 5th group the most deprived.

[d]Frequency of alcohol consumption was only collected from current alcohol drinkers.
