# Supplementary material for: Sex differences in risk factors for incident peripheral artery disease hospitalisation or death: Cohort study of UK Biobank participants
Source: PLoS One. 2023 Oct 18;18(10):e0292083. doi: 10.1371/journal.pone.0292083 (PMC10584119; doi:10.1371/journal.pone.0292083)
Supplement: S8 Table — (PDF) [file pone.0292083.s014.pdf]

S8 Table. Sex-specific hazard ratios and women-to-men ratio of hazard ratios for risk factors in an additional sensitivity analysis.

Risk factors	Age-adjusted			Multivariable-adjusted		
	HR (95% CI)		Women-to-men ratio of HRs (95% CI)	HR (95% CI)		Women-to-men ratio of HRs (95% CI)
	Women	Men		Women	Men	
Systolic blood pressure, per 10 mmHg	1.10 (1.07, 1.12)	1.06 (1.04, 1.07)	1.03 (1.01, 1.06)	1.10 (1.08, 1.13)	1.08 (1.06, 1.10)	1.02 (0.99, 1.05)
Diastolic blood pressure, per 5 mmHg	0.97 (0.95, 0.99)	0.93 (0.92, 0.95)	1.04 (1.02, 1.07)	0.98 (0.96, 1.00)	0.96 (0.95, 0.98)	1.02 (0.99, 1.05)
Pulse pressure, per 5 mmHg	1.09 (1.08, 1.11)	1.09 (1.08, 1.10)	1.00 (0.99, 1.02)	1.09 (1.07, 1.10)	1.08 (1.07, 1.09)	1.00 (0.99, 1.02)
AHA hypertension categories						
Normal	Reference	Reference	Reference	Reference	Reference	Reference
Elevated	0.96 (0.80, 1.14)	1.00 (0.87, 1.15)	0.99 (0.80, 1.22)	0.96 (0.80, 1.16)	1.06 (0.91, 1.24)	0.91 (0.71, 1.16)
Stage 1 hypertension	1.06 (0.91, 1.23)	0.91 (0.80, 1.02)	1.16 (0.97, 1.40)	1.05 (0.90, 1.23)	1.02 (0.89, 1.17)	1.03 (0.84, 1.27)
Stage 2 hypertension	1.39 (1.22, 1.59)	1.05 (0.94, 1.18)	1.31 (1.10, 1.55)	1.34 (1.16, 1.55)	1.17 (1.03, 1.33)	1.15 (0.94, 1.39)
Smoking status						
Never	Reference	Reference	Reference	Reference	Reference	Reference
Former	1.74 (1.58, 1.91)	2.06 (1.92, 2.21)	0.81 (0.72, 0.91)	1.67 (1.52, 1.83)	2.05 (1.91, 2.21)	0.81 (0.72, 0.92)
Current	6.22 (5.61, 6.89)	5.07 (4.69, 5.48)	1.17 (1.03, 1.32)	5.33 (4.79, 5.92)	4.48 (4.13, 4.88)	1.19 (1.04, 1.36)
Former versus current smokers	0.28 (0.25, 0.31)	0.40 (0.37, 0.43)	0.70 (0.62, 0.78)	0.31 (0.28, 0.35)	0.45 (0.42, 0.49)	0.69 (0.61, 0.78)
Current versus non-current smokers	4.91 (4.48, 5.38)	3.42 (3.20, 3.65)	1.44 (1.28, 1.61)	4.19 (3.83, 4.59)	2.94 (2.76, 3.14)	1.43 (1.28, 1.59)
By smoking intensity[a]						
Never	Reference	Reference	Reference	Reference	Reference	Reference
≤9 cigarettes per day	3.68 (2.89, 4.69)	3.53 (2.84, 4.38)	1.11 (0.82, 1.51)	3.39 (2.66, 4.33)	3.09 (2.45, 3.89)	1.10 (0.78, 1.54)
10-19 cigarettes per day	7.00 (6.08, 8.05)	6.24 (5.56, 7.00)	1.05 (0.88, 1.25)	6.08 (5.27, 7.03)	5.44 (4.81, 6.16)	1.12 (0.92, 1.35)
≥20 cigarettes per day	9.82 (8.47, 11.40)	8.13 (7.34, 9.02)	1.14 (0.96, 1.36)	8.27 (7.09, 9.65)	6.88 (6.14, 7.70)	1.20 (0.99, 1.46)
No diabetes	Reference	Reference	Reference	Reference	Reference	Reference
Type 1 diabetes	14.83 (0.39, 21.16)	7.18 (5.46, 9.44)	1.72 (1.09, 2.70)	6.79 (4.51, 10.23)	4.76 (3.51, 6.45)	1.43 (0.86, 2.38)
Type 2 diabetes[b]	4.28 (3.83, 4.78)	3.97 (3.72, 4.24)	0.99 (0.87, 1.12)	2.13 (1.86, 2.44)	2.39 (2.20, 2.59)	0.89 (0.76, 1.04)
Cholesterol, per 1 mmol/L						
Total cholesterol	0.77 (0.74, 0.80)	0.75 (0.73, 0.77)	1.05 (1.01, 1.10)	0.99 (0.95, 1.03)	1.00 (0.97, 1.04)	0.99 (0.93, 1.04)
HDL-C	0.33 (0.29, 0.38)	0.38 (0.34, 0.42)	0.89 (0.75, 1.05)	0.69 (0.60, 0.79)	0.82 (0.73, 0.92)	0.84 (0.70, 1.00)
LDL-C	0.76 (0.72, 0.80)	0.69 (0.66, 0.71)	1.14 (1.07, 1.21)	1.03 (0.97, 1.08)	1.01 (0.97, 1.05)	1.02 (0.95, 1.09)

Risk factors	Age-adjusted			Multivariable-adjusted		
	HR (95% CI)		Women-to-men ratio of HRs (95% CI)	HR (95% CI)		Women-to-men ratio of HRs (95% CI)
	Women	Men		Women	Men	
Total cholesterol						
Normal (<6.2 mmol/L)	Reference	Reference	Reference	Reference	Reference	Reference
Elevated (≥6.2 mmol/L)	0.70 (0.64, 0.77)	0.66 (0.61, 0.71)	1.08 (0.97, 1.21)	1.06 (0.96, 1.18)	0.99 (0.91, 1.08)	1.08 (0.94, 1.23)
HDL-C categories						
≤1.03	2.49 (2.16, 2.88)	1.93 (1.80, 2.07)	1.29 (1.10, 1.51)	1.48 (1.27, 1.73)	1.25 (1.16, 1.34)	1.19 (1.00, 1.41)
>1.03 and ≤1.55	Reference	Reference	Reference	Reference	Reference	Reference
>1.55 and ≤2.07	0.64 (0.58, 0.71)	0.73 (0.65, 0.81)	0.88 (0.76, 1.03)	0.87 (0.78, 0.97)	0.92 (0.82, 1.02)	0.95 (0.82, 1.11)
>2.07	0.53 (0.45, 0.63)	1.49 (1.22, 1.82)	0.36 (0.27, 0.46)	0.80 (0.67, 0.97)	1.65 (1.34, 2.02)	0.49 (0.37, 0.64)
Body mass index, per 5 kg/m^2	1.32 (1.27, 1.36)	1.38 (1.34, 1.42)	0.94 (0.90, 0.98)	1.29 (1.25, 1.34)	1.37 (1.33, 1.41)	0.94 (0.90, 0.99)
Body mass index (kg/m^2) categories						
Underweight (<18.5)	2.64 (1.86, 3.74)	2.8 (1.82, 4.31)	0.92 (0.53, 1.58)	1.94 (1.35, 2.78)	1.79 (1.14, 2.83)	1.08 (0.60, 1.93)
Healthy weight (18.5-24.9)	Reference	Reference	Reference	Reference	Reference	Reference
Overweight (25-29.9)	1.15 (1.04, 1.27)	0.96 (0.89, 1.04)	1.22 (1.08, 1.38)	1.15 (1.04, 1.27)	0.97 (0.90, 1.05)	1.18 (1.03, 1.34)
Obese (30 and above)	1.87 (1.69, 2.07)	1.78 (1.65, 1.92)	1.01 (0.89, 1.14)	1.77 (1.60, 1.97)	1.75 (1.62, 1.90)	1.01 (0.89, 1.15)
Waist circumference, per 10 cm	1.39 (1.35, 1.43)	1.34 (1.31, 1.37)	1.01 (0.98, 1.05)	1.33 (1.29, 1.37)	1.32 (1.29, 1.36)	1.01 (0.97, 1.04)
Waist-to-hip ratio, per 0.1	1.45 (1.42, 1.48)	1.72 (1.67, 1.77)	0.84 (0.81, 0.87)	1.43 (1.39, 1.46)	1.70 (1.64, 1.76)	0.84 (0.81, 0.88)
Waist-to-height ratio, per 0.1	1.69 (1.61, 1.77)	1.71 (1.65, 1.78)	0.96 (0.90, 1.01)	1.59 (1.51, 1.66)	1.66 (1.59, 1.73)	0.96 (0.90, 1.02)
History of stroke	3.70 (3.09, 4.44)	2.93 (2.62, 3.28)	1.25 (1.02, 1.54)	3.36 (2.80, 4.03)	2.69 (2.40, 3.02)	1.25 (1.01, 1.55)
History of myocardial infarction	5.55 (4.68, 6.58)	3.38 (3.12, 3.66)	1.59 (1.33, 1.92)	4.83 (4.07, 5.73)	3.28 (3.02, 3.55)	1.47 (1.22, 1.78)
Socioeconomic status[c]						
1st (least deprived)	Reference	Reference	Reference	Reference	Reference	Reference
2nd	1.18 (1.05, 1.33)	1.25 (1.15, 1.35)	0.91 (0.79, 1.04)	1.05 (0.92, 1.19)	1.14 (1.04, 1.25)	0.92 (0.78, 1.08)
3rd	1.49 (1.31, 1.69)	1.45 (1.32, 1.58)	1.01 (0.87, 1.17)	1.20 (1.05, 1.38)	1.26 (1.14, 1.39)	0.95 (0.81, 1.13)
4th	1.83 (1.61, 2.07)	1.67 (1.52, 1.82)	1.03 (0.89, 1.20)	1.36 (1.19, 1.55)	1.32 (1.20, 1.46)	1.03 (0.87, 1.21)
5th (most deprived)	2.70 (2.41, 3.03)	2.79 (2.58, 3.01)	0.90 (0.79, 1.03)	1.60 (1.41, 1.81)	1.83 (1.68, 2.00)	0.87 (0.75, 1.02)
eGFRcys, per 10 $ml/min/1.73m^2$	0.69 (0.68, 0.71)	0.73 (0.71, 0.74)	0.96 (0.93, 0.99)	0.80 (0.78, 0.83)	0.82 (0.80, 0.83)	0.99 (0.95, 1.02)
eGFRcys ($ml/min/1.73m^2$) categories						
Normal or high (≥90)	Reference	Reference	Reference	Reference	Reference	Reference

Risk factors	Age-adjusted			Multivariable-adjusted		
	HR (95% CI)		Women-to-men ratio of HRs (95% CI)	HR (95% CI)		Women-to-men ratio of HRs (95% CI)
	Women	Men		Women	Men	
Decreased (<90)	1.91 (1.72, 2.12)	1.82 (1.70, 1.96)	1.04 (0.92, 1.17)	1.36 (1.21, 1.51)	1.49 (1.38, 1.61)	0.91 (0.79, 1.04)
C-reactive protein, per 1 mg/L	1.23 (1.21, 1.26)	1.20 (1.18, 1.22)	1.03 (1.00, 1.05)	1.15 (1.11, 1.18)	1.15 (1.12, 1.17)	1.00 (0.96, 1.03)
Alcohol drinker status						
Never	Reference	Reference	Reference	Reference	Reference	Reference
Previous	1.55 (1.27, 1.89)	1.59 (1.31, 1.94)	0.97 (0.74, 1.28)	1.10 (0.90, 1.34)	1.04 (0.85, 1.27)	1.05 (0.79, 1.40)
Current	0.68 (0.59, 0.78)	0.79 (0.67, 0.93)	0.86 (0.69, 1.07)	0.61 (0.53, 0.70)	0.67 (0.57, 0.79)	0.91 (0.73, 1.14)
Frequency of alcohol consumption[d]						
Never	Reference	Reference	Reference	Reference	Reference	Reference
Special occasions only	1.03 (0.87, 1.20)	1.17 (0.98, 1.41)	0.87 (0.68, 1.12)	0.85 (0.72, 1.00)	0.92 (0.76, 1.11)	0.93 (0.72, 1.18)
One to three times a month	0.76 (0.64, 0.91)	0.89 (0.74, 1.08)	0.86 (0.66, 1.11)	0.68 (0.56, 0.81)	0.75 (0.62, 0.91)	0.90 (0.69, 1.17)
Once or twice a week	0.58 (0.49, 0.68)	0.78 (0.66, 0.93)	0.74 (0.58, 0.94)	0.53 (0.45, 0.63)	0.68 (0.57, 0.81)	0.78 (0.62, 1.00)
Three or four times a week	0.50 (0.42, 0.60)	0.62 (0.52, 0.74)	0.80 (0.63, 1.03)	0.47 (0.39, 0.56)	0.55 (0.46, 0.66)	0.85 (0.66, 1.10)
Daily or almost daily	0.64 (0.54, 0.75)	0.81 (0.68, 0.96)	0.79 (0.62, 1.00)	0.52 (0.43, 0.62)	0.65 (0.54, 0.77)	0.80 (0.63, 1.03)

In this sensitivity analysis, only diagnostic codes for peripheral artery disease (S1 Table) were used.

AHA denotes American Heart Association, CI confidence interval, eGFRcys estimated Glomerular Filtration Rate calculated using cystatin C, HDL high-density lipoprotein, HR hazard ratio, LDL low-density lipoprotein.

[a]Smoking intensity was only collected from current smokers.

[b]Defined as diagnosis before the age of 30 years old and receiving insulin treatment.

[c] Socioeconomic status was determined using the Townsend Deprivation Index and grouped into five groups based on the cut-offs for the UK national equal fifths, with the 1st group containing the least socially deprived and the 5th group the most deprived.

[d]Frequency of alcohol consumption was only collected from current alcohol drinkers.
