# Supplementary material for: Sex differences in risk factors for incident peripheral artery disease hospitalisation or death: Cohort study of UK Biobank participants
Source: PLoS One. 2023 Oct 18;18(10):e0292083. doi: 10.1371/journal.pone.0292083 (PMC10584119; doi:10.1371/journal.pone.0292083)
Supplement: S10 Table — (PDF) [file pone.0292083.s016.pdf]

S10 Table. Sex-specific multivariable-adjusted hazard ratios and women-to-men ratio of hazard ratios for risk factors by age group.

**Risk factors (higher continuous variables or by category for categorical variables)**	**Age subgroup (years old)**	**Women**		**Men**		**Women-to-men**	
		**HR (95% CI)**	**P value**	**HR (95% CI)**	**P value**	**Ratio of HRs (95% CI)**	**P value**
Systolic blood pressure, per 10 mmHg	<60	1.16 (1.11, 1.21)	0.002	1.13 (1.09, 1.17)	<0.001	1.03 (0.97, 1.08)	0.80
	≥60	1.08 (1.05, 1.10)		1.05 (1.03, 1.07)		1.02 (0.99, 1.05)	
Diastolic blood pressure, per 5 mmHg	<60	1.03 (0.99, 1.07)	0.003	1.01 (0.98, 1.04)	<0.001	1.02 (0.97, 1.07)	0.61
	≥60	0.96 (0.94, 0.99)		0.95 (0.93, 0.97)		1.01 (0.98, 1.05)	
Pulse pressure, per 5 mmHg	<60	1.12 (1.10, 1.15)	0.002	1.12 (1.09, 1.14)	<0.001	1.01 (0.97, 1.04)	0.51
	≥60	1.07 (1.06, 1.09)		1.07 (1.05, 1.08)		1.01 (0.99, 1.02)	
AHA hypertension categories			0.52		0.03		0.30
Elevated versus normal	<60	0.89 (0.66, 1.19)	0.33	1.26 (0.98, 1.62)	0.09	0.70 (0.48, 1.03)	0.06
	≥60	1.07 (0.85, 1.33)		0.97 (0.81, 1.16)		1.10 (0.83, 1.47)	
Stage 1 hypertension versus normal	<60	1.12 (0.89, 1.41)	0.50	1.16 (0.93, 1.45)	0.11	0.97 (0.70, 1.33)	0.58
	≥60	1.01 (0.82, 1.23)		0.93 (0.80, 1.09)		1.08 (0.84, 1.40)	
Stage 2 hypertension versus normal	<60	1.43 (1.15, 1.78)	0.55	1.43 (1.16, 1.76)	0.01	1.00 (0.74, 1.36)	0.17
	≥60	1.31 (1.09, 1.57)		1.03 (0.89, 1.20)		1.27 (1.00, 1.60)	
Smoking status			0.70		0.42		0.73
Former versus never smokers	<60	1.42 (1.19, 1.69)	0.08	1.53 (1.34, 1.75)	<0.001	0.92 (0.74, 1.15)	0.22
	≥60	1.70 (1.53, 1.88)		2.18 (2.00, 2.37)		0.78 (0.68, 0.89)	
Current versus never smokers	<60	4.82 (4.07, 5.70)	0.34	4.45 (3.91, 5.06)	0.55	1.08 (0.88, 1.34)	0.55
	≥60	5.32 (4.70, 6.03)		4.24 (3.83, 4.70)		1.25 (1.07, 1.47)	
Former versus current smokers	<60	0.29 (0.24, 0.35)	0.41	0.33 (0.29, 0.38)	<0.001	0.87 (0.70, 1.09)	0.07
	≥60	0.32 (0.28, 0.36)		0.52 (0.47, 0.56)		0.62 (0.53, 0.72)	
Current versus non-current smokers	<60	4.21 (3.62, 4.90)	0.90	3.67 (3.29, 4.09)	<0.001	1.15 (0.95, 1.38)	0.02
	≥60	4.16 (3.72, 4.65)		2.58 (2.38, 2.80)		1.61 (1.40, 1.85)	
By smoking intensity[a]			0.43		0.41		0.51
≤9 cigarettes per day versus never	<60	2.90 (1.95, 4.31)	0.15	2.91 (2.06, 4.11)	0.57	1.00 (0.59, 1.69)	0.63
	≥60	4.11 (3.16, 5.36)		3.32 (2.51, 4.41)		1.24 (0.84, 1.82)	
10-19 cigarettes per day versus never	<60	5.43 (4.31, 6.82)	0.42	4.75 (3.92, 5.74)	0.11	1.14 (0.85, 1.54)	0.44

	≥60	6.12 (5.15, 7.27)		5.81 (5.00, 6.75)		1.05 (0.84, 1.32)	
≥20 cigarettes per day versus never	<60	7.93 (6.30, 9.99)	0.89	7.37 (6.28, 8.65)	0.06	1.08 (0.81, 1.42)	0.41
	≥60	8.11 (6.70, 9.83)		6.01 (5.17, 6.98)		1.35 (1.06, 1.72)	
Diabetes			<0.001		<0.001		0.26
Type 1 diabetes versus no diabetes	<60	4.31 (2.27, 8.19)	0.21	4.51 (2.92, 6.97)	0.98	0.96 (0.44, 2.07)	0.46
	≥60	7.35 (4.31, 12.52)		4.55 (3.01, 6.87)		1.61 (0.82, 3.17)	
Type 2 diabetes[b] versus no diabetes	<60	3.25 (2.54, 4.16)	<0.001	3.34 (2.85, 3.91)	<0.001	0.97 (0.73, 1.30)	0.24
	≥60	1.65 (1.40, 1.93)		2.00 (1.82, 2.19)		0.82 (0.69, 0.99)	
Cholesterol, per 1 mmol/L							
Total cholesterol	<60	1.04 (0.97, 1.12)	0.11	1.02 (0.97, 1.08)	0.44	1.02 (0.93, 1.11)	0.75
	≥60	0.97 (0.93, 1.02)		1.00 (0.96, 1.03)		0.98 (0.92, 1.04)	
HDL-C	<60	0.70 (0.55, 0.90)	0.60	0.78 (0.63, 0.96)	0.59	0.90 (0.65, 1.24)	0.94
	≥60	0.65 (0.56, 0.76)		0.83 (0.73, 0.95)		0.78 (0.64, 0.95)	
LDL-C	<60	1.10 (1.01, 1.20)	0.12	1.01 (0.94, 1.09)	0.98	1.09 (0.97, 1.22)	0.37
	≥60	1.01 (0.95, 1.07)		1.01 (0.96, 1.07)		1.00 (0.92, 1.08)	
Elevated (≥6.2 mmol/L) versus normal total cholesterol	<60	1.10 (0.93, 1.31)	0.41	1.04 (0.91, 1.20)	0.58	1.06 (0.85, 1.31)	0.96
	≥60	1.01 (0.90, 1.13)		0.99 (0.89, 1.10)		1.02 (0.87, 1.19)	
HDL-C categories (versus >1.55 and ≤2.07)			0.79		0.94		0.80
≤1.03	<60	1.39 (1.07, 1.79)	0.46	1.29 (1.13, 1.47)	0.65	1.07 (0.81, 1.43)	0.59
	≥60	1.56 (1.31, 1.86)		1.25 (1.15, 1.35)		1.25 (1.03, 1.52)	
>1.03 and ≤1.55	<60	0.82 (0.67, 0.99)	0.66	0.86 (0.70, 1.06)	0.41	0.95 (0.72, 1.26)	0.94
	≥60	0.86 (0.76, 0.97)		0.95 (0.84, 1.07)		0.91 (0.76, 1.07)	
>2.07	<60	0.90 (0.65, 1.23)	0.35	1.72 (1.19, 2.48)	0.60	0.52 (0.32, 0.84)	0.87
	≥60	0.75 (0.61, 0.92)		1.53 (1.21, 1.94)		0.49 (0.36, 0.67)	
Body mass index, per 5 kg/m^2	<60	1.30 (1.23, 1.37)	0.25	1.33 (1.26, 1.40)	0.79	0.98 (0.91, 1.05)	0.15
	≥60	1.25 (1.20, 1.30)		1.34 (1.29, 1.39)		0.93 (0.88, 0.99)	
Body mass index (kg/m^2) categories			0.16		0.53		0.09
Underweight (<18.5) versus healthy weight (18.5-24.9)	<60	1.83 (1.00, 3.36)	0.87	1.44 (0.74, 2.79)	0.45	1.27 (0.52, 3.13)	0.68
	≥60	1.94 (1.28, 2.96)		2.02 (1.14, 3.58)		0.96 (0.47, 1.95)	
Overweight (25-29.9) versus healthy weight (18.5-24.9)	<60	1.25 (1.04, 1.49)	0.43	0.86 (0.75, 0.98)	0.03	1.45 (1.16, 1.82)	0.05
	≥60	1.14 (1.02, 1.28)		1.03 (0.94, 1.13)		1.11 (0.96, 1.29)	

Obese (≥30) versus healthy weight (18.5-24.9)	<60	1.90 (1.60, 2.27)	0.16	1.65 (1.44, 1.89)	0.80	1.15 (0.92, 1.44)	0.13
	≥60	1.64 (1.45, 1.84)		1.69 (1.54, 1.86)		0.97 (0.83, 1.13)	
Waist circumference, per 10 cm	<60	1.36 (1.30, 1.42)	0.08	1.30 (1.25, 1.36)	0.68	1.04 (0.98, 1.10)	0.20
	≥60	1.29 (1.24, 1.33)		1.29 (1.26, 1.33)		1.00 (0.95, 1.04)	
Waist-to-hip ratio, per 0.1	<60	1.39 (1.34, 1.44)	<0.001	1.62 (1.53, 1.70)	0.53	0.86 (0.80, 0.91)	0.03
	≥60	1.59 (1.50, 1.70)		1.66 (1.58, 1.74)		0.96 (0.89, 1.04)	
Waist-to-height ratio, per 0.1	<60	1.62 (1.50, 1.74)	0.15	1.62 (1.52, 1.74)	0.53	1.00 (0.90, 1.10)	0.45
	≥60	1.51 (1.43, 1.60)		1.58 (1.51, 1.66)		0.96 (0.89, 1.03)	
History of stroke versus no	<60	4.33 (3.03, 6.18)	0.11	4.03 (3.22, 5.04)	<0.001	1.07 (0.70, 1.63)	0.38
	≥60	3.09 (2.53, 3.78)		2.38 (2.09, 2.71)		1.30 (1.02, 1.65)	
History of myocardial infarction versus no	<60	5.96 (4.02, 8.84)	0.21	4.57 (3.87, 5.38)	<0.001	1.31 (0.85, 2.00)	0.38
	≥60	4.52 (3.76, 5.42)		2.87 (2.62, 3.14)		1.57 (1.28, 1.93)	
Socioeconomic status[c]			0.08		0.13		0.38
2nd versus 1st	<60	1.10 (0.86, 1.41)	0.44	1.29 (1.08, 1.54)	0.11	0.85 (0.63, 1.15)	0.78
	≥60	0.99 (0.86, 1.14)		1.09 (0.99, 1.21)		0.90 (0.76, 1.07)	
3rd versus 1st	<60	1.35 (1.05, 1.72)	0.27	1.44 (1.20, 1.72)	0.07	0.94 (0.69, 1.27)	0.94
	≥60	1.15 (0.99, 1.33)		1.18 (1.05, 1.32)		0.97 (0.81, 1.17)	
4th versus 1st	<60	1.47 (1.16, 1.87)	0.21	1.50 (1.25, 1.79)	0.05	0.98 (0.73, 1.32)	0.99
	≥60	1.23 (1.05, 1.43)		1.21 (1.08, 1.36)		1.01 (0.84, 1.22)	
5th versus 1st	<60	1.72 (1.38, 2.15)	0.11	1.90 (1.62, 2.22)	0.19	0.91 (0.69, 1.19)	0.39
	≥60	1.39 (1.20, 1.60)		1.68 (1.52, 1.85)		0.83 (0.70, 0.99)	
eGFRcys, per 10 ml/min/1.73m^2	<60	0.77 (0.73, 0.81)	0.08	0.81 (0.79, 0.84)	0.70	0.95 (0.89, 1.01)	0.54
	≥60	0.81 (0.79, 0.84)		0.82 (0.80, 0.84)		0.99 (0.95, 1.03)	
Decreased eGFRcys (<90 ml/min/1.73m^2) versus normal or high (≥90)	<60	1.58 (1.33, 1.87)	0.06	1.61 (1.43, 1.81)	0.06	0.98 (0.80, 1.20)	0.95
	≥60	1.28 (1.12, 1.46)		1.40 (1.27, 1.53)		0.92 (0.78, 1.08)	
C-reactive protein, per 1 mg/L	<60	1.18 (1.12, 1.24)	0.26	1.17 (1.13, 1.21)	0.09	1.01 (0.94, 1.07)	0.91
	≥60	1.14 (1.10, 1.17)		1.13 (1.10, 1.15)		1.01 (0.97, 1.05)	
Alcohol drinker status			0.14		0.70		0.49
Previous versus never	<60	1.19 (0.84, 1.69)	0.72	1.42 (1.00, 2.02)	0.08	0.84 (0.51, 1.37)	0.39
	≥60	1.10 (0.88, 1.39)		0.97 (0.77, 1.24)		1.13 (0.81, 1.58)	
Current versus never	<60	0.57 (0.43, 0.74)	0.37	0.79 (0.58, 1.07)	0.45	0.72 (0.48, 1.07)	0.29
	≥60	0.65 (0.56, 0.77)		0.69 (0.57, 0.84)		0.95 (0.74, 1.23)	

Frequency of alcohol consumption[d]			0.02		0.98		0.09
Special occasions only versus never	<60	0.85 (0.63, 1.14)	0.89	1.09 (0.78, 1.53)	0.41	0.78 (0.50, 1.22)	0.56
	≥60	0.87 (0.73, 1.05)		0.93 (0.74, 1.15)		0.94 (0.71, 1.25)	
One to three times a month versus never	<60	0.60 (0.44, 0.83)	0.39	0.93 (0.66, 1.30)	0.28	0.65 (0.41, 1.03)	0.22
	≥60	0.71 (0.58, 0.87)		0.74 (0.59, 0.93)		0.96 (0.71, 1.3)	
Once or twice a week versus never	<60	0.52 (0.38, 0.69)	0.53	0.81 (0.59, 1.11)	0.48	0.64 (0.41, 0.99)	0.46
	≥60	0.58 (0.48, 0.69)		0.70 (0.58, 0.86)		0.82 (0.62, 1.08)	
Three or four times a week versus never	<60	0.38 (0.27, 0.52)	0.07	0.54 (0.39, 0.75)	0.55	0.70 (0.44, 1.11)	0.51
	≥60	0.54 (0.44, 0.66)		0.61 (0.49, 0.74)		0.89 (0.67, 1.18)	
Daily or almost daily versus never	<60	0.44 (0.32, 0.62)	0.19	0.83 (0.60, 1.13)	0.21	0.54 (0.34, 0.85)	0.09
	≥60	0.57 (0.47, 0.70)		0.65 (0.53, 0.79)		0.89 (0.67, 1.17)	

AHA denotes American Heart Association, CI confidence interval, eGFRcys estimated Glomerular Filtration Rate calculated using cystatin C, HDL high-density lipoprotein, HR hazard ratio, LDL low-density lipoprotein.

[a]Smoking intensity was only collected from current smokers.

[b]Defined as diagnosis before the age of 30 years old and receiving insulin treatment.

[c]S Socioeconomic status was determined using the Townsend Deprivation Index and grouped into five groups based on the cut-offs for the UK national equal fifths, with the 1st group containing the least socially deprived and the 5th group the most deprived.

[d]Frequency of alcohol consumption was only collected from current alcohol drinkers.
