# Supplementary material for: Sex differences in risk factors for incident peripheral artery disease hospitalisation or death: Cohort study of UK Biobank participants
Source: PLoS One. 2023 Oct 18;18(10):e0292083. doi: 10.1371/journal.pone.0292083 (PMC10584119; doi:10.1371/journal.pone.0292083)
Supplement: S11 Table — (PDF) [file pone.0292083.s017.pdf]

S11 Table. Sex-specific multivariable-adjusted hazard ratios and women-to-men ratio of hazard ratios for risk factors by smoking status.

**Risk factors (higher continuous variables or by category for categorical variables)**	**Smoking status**	**Women**		**Men**		**Women-to-men**	
		**HR (95% CI)**	**P value**	**HR (95% CI)**	**P value**	**Ratio of HRs (95% CI)**	**P value**
Systolic blood pressure, per 10 mmHg	Never	1.08 (1.04, 1.12)	0.24	1.05 (1.01, 1.09)	0.12	1.03 (0.98, 1.08)	0.70
	Ever	1.11 (1.08, 1.14)		1.08 (1.06, 1.10)		1.03 (0.99, 1.06)	
Diastolic blood pressure, per 5 mmHg	Never	1.00 (0.96, 1.03)	0.31	0.98 (0.95, 1.01)	0.48	1.02 (0.97, 1.07)	0.63
	Ever	0.98 (0.95, 1.00)		0.97 (0.95, 0.98)		1.01 (0.98, 1.04)	
Pulse pressure, per 5 mmHg	Never	1.07 (1.04, 1.09)	0.03	1.05 (1.03, 1.08)	0.03	1.01 (0.98, 1.04)	0.92
	Ever	1.10 (1.08, 1.12)		1.08 (1.07, 1.10)		1.01 (0.99, 1.03)	
AHA hypertension categories			0.44		0.14		0.59
Elevated versus normal	Never	1.01 (0.75, 1.35)	0.93	1.07 (0.81, 1.42)	0.88	0.94 (0.62, 1.41)	0.89
	Ever	1.02 (0.82, 1.27)		1.05 (0.88, 1.24)		0.98 (0.74, 1.28)	
Stage 1 hypertension versus normal	Never	1.00 (0.77, 1.29)	0.62	0.85 (0.66, 1.10)	0.16	1.17 (0.81, 1.67)	0.55
	Ever	1.08 (0.90, 1.30)		1.05 (0.91, 1.22)		1.02 (0.81, 1.30)	
Stage 2 hypertension versus normal	Never	1.26 (1.00, 1.60)	0.46	1.01 (0.80, 1.28)	0.25	1.24 (0.89, 1.74)	0.71
	Ever	1.41 (1.19, 1.67)		1.19 (1.04, 1.37)		1.18 (0.95, 1.47)	
Diabetes			0.29		<0.001		0.02
Type 1 diabetes versus no diabetes	Never	8.71 (4.96, 15.32)	0.09	5.56 (3.31, 9.34)	0.40	1.57 (0.73, 3.37)	0.43
	Ever	4.27 (2.34, 7.77)		4.20 (2.92, 6.05)		1.01 (0.50, 2.05)	
Type 2 diabetes[a] versus no diabetes	Never	2.19 (1.76, 2.72)	0.34	3.17 (2.70, 3.72)	<0.001	0.69 (0.53, 0.91)	0.01
	Ever	1.90 (1.61, 2.25)		2.07 (1.89, 2.26)		0.92 (0.76, 1.11)	
Cholesterol, per 1 mmol/L							
Total cholesterol	Never	0.98 (0.92, 1.05)	0.68	0.99 (0.93, 1.06)	0.65	0.99 (0.90, 1.08)	0.18
	Ever	1.00 (0.95, 1.05)		1.01 (0.98, 1.05)		0.99 (0.93, 1.05)	
HDL-C	Never	0.69 (0.56, 0.86)	0.58	1.03 (0.82, 1.30)	0.02	0.67 (0.49, 0.92)	1.00
	Ever	0.64 (0.54, 0.76)		0.76 (0.67, 0.86)		0.84 (0.69, 1.04)	
LDL-C	Never	1.01 (0.93, 1.10)	0.50	0.99 (0.91, 1.08)	0.50	1.02 (0.91, 1.15)	0.24
	Ever	1.05 (0.99, 1.12)		1.03 (0.98, 1.07)		1.02 (0.95, 1.11)	
Elevated (≥6.2 mmol/L) versus normal total cholesterol	Never	1.01 (0.87, 1.18)	0.68	1.00 (0.85, 1.18)	0.85	1.01 (0.80, 1.27)	0.48
	Ever	1.05 (0.94, 1.19)		1.02 (0.93, 1.12)		1.03 (0.89, 1.21)	

HDL-C categories (versus >1.55 and ≤2.07)			0.28		0.54		0.52
≤1.03	Never	1.67 (1.31, 2.14)	0.34	1.09 (0.94, 1.27)	0.03	1.53 (1.15, 2.03)	0.29
	Ever	1.44 (1.21, 1.73)		1.32 (1.22, 1.43)		1.09 (0.9, 1.33)	
>1.03 and ≤1.55	Never	0.89 (0.76, 1.05)	0.46	1.14 (0.93, 1.39)	0.03	0.79 (0.61, 1.02)	0.58
	Ever	0.82 (0.72, 0.94)		0.87 (0.77, 0.98)		0.95 (0.79, 1.13)	
>2.07	Never	0.89 (0.68, 1.18)	0.27	1.69 (1.06, 2.67)	0.74	0.53 (0.31, 0.91)	0.47
	Ever	0.73 (0.58, 0.91)		1.54 (1.23, 1.91)		0.47 (0.35, 0.65)	
Body mass index, per 5 kg/m^2	Never	1.37 (1.30, 1.44)	<0.001	1.51 (1.43, 1.60)	<0.001	0.91 (0.84, 0.98)	0.36
	Ever	1.21 (1.16, 1.26)		1.27 (1.23, 1.32)		0.95 (0.90, 1.00)	
Body mass index (kg/m^2) categories			0.01		<0.001		0.56
Underweight (<18.5) versus healthy weight (18.5-24.9)	Never	1.41 (0.67, 3.00)	0.37	1.11 (0.28, 4.48)	0.48	1.27 (0.26, 6.17)	0.96
	Ever	2.07 (1.40, 3.06)		1.88 (1.19, 2.97)		1.10 (0.60, 2.00)	
Overweight (25-29.9) versus healthy weight (18.5-24.9)	Never	1.30 (1.10, 1.52)	0.14	1.01 (0.86, 1.17)	0.58	1.29 (1.03, 1.61)	0.36
	Ever	1.11 (0.99, 1.26)		0.96 (0.88, 1.04)		1.16 (1.00, 1.35)	
Obese (≥30) versus healthy weight (18.5-24.9)	Never	2.01 (1.71, 2.37)	0.01	2.24 (1.92, 2.60)	<0.001	0.90 (0.72, 1.13)	0.39
	Ever	1.56 (1.38, 1.77)		1.51 (1.38, 1.66)		1.03 (0.88, 1.20)	
Waist circumference, per 10 cm	Never	1.39 (1.33, 1.46)	0.001	1.42 (1.36, 1.49)	<0.001	0.98 (0.92, 1.04)	0.32
	Ever	1.27 (1.22, 1.31)		1.25 (1.22, 1.29)		1.01 (0.97, 1.06)	
Waist-to-hip ratio, per 0.1	Never	1.68 (1.54, 1.83)	<0.001	1.66 (1.58, 1.75)	0.36	1.01 (0.92, 1.11)	0.01
	Ever	1.39 (1.35, 1.44)		1.61 (1.53, 1.69)		0.87 (0.82, 0.92)	
Waist-to-height ratio, per 0.1	Never	1.69 (1.57, 1.81)	0.003	1.85 (1.72, 2.00)	<0.001	0.91 (0.82, 1.01)	0.25
	Ever	1.47 (1.39, 1.55)		1.51 (1.44, 1.58)		0.97 (0.90, 1.05)	
History of stroke versus no	Never	4.06 (3.07, 5.38)	0.07	3.56 (2.83, 4.48)	0.001	1.14 (0.79, 1.64)	0.63
	Ever	2.91 (2.32, 3.64)		2.29 (2.02, 2.61)		1.27 (0.98, 1.64)	
History of myocardial infarction versus no	Never	3.82 (2.69, 5.41)	0.35	3.05 (2.51, 3.69)	0.55	1.25 (0.84, 1.87)	0.27
	Ever	4.58 (3.79, 5.53)		2.85 (2.61, 3.11)		1.61 (1.30, 1.98)	
Socioeconomic status[b]			0.12		0.54		0.08
2nd versus 1st	Never	1.00 (0.83, 1.21)	0.81	1.05 (0.89, 1.24)	0.29	0.95 (0.74, 1.23)	0.74
	Ever	1.03 (0.88, 1.21)		1.17 (1.05, 1.29)		0.89 (0.73, 1.07)	
3rd versus 1st	Never	1.13 (0.92, 1.38)	0.44	1.04 (0.86, 1.26)	0.04	1.08 (0.82, 1.43)	0.67
	Ever	1.25 (1.06, 1.47)		1.30 (1.17, 1.45)		0.96 (0.78, 1.16)	

4th versus 1st	Never	1.16 (0.94, 1.44)	0.20	1.33 (1.11, 1.61)	0.64	0.87 (0.66, 1.15)	0.08
	Ever	1.38 (1.17, 1.62)		1.26 (1.13, 1.41)		1.09 (0.90, 1.33)	
5th versus 1st	Never	1.37 (1.12, 1.68)	0.27	1.64 (1.38, 1.95)	0.42	0.84 (0.64, 1.10)	0.21
	Ever	1.57 (1.36, 1.82)		1.77 (1.61, 1.94)		0.89 (0.75, 1.06)	
eGFRcys, per 10 ml/min/1.73m^2	Never	0.77 (0.74, 0.81)	0.07	0.80 (0.77, 0.83)	0.12	0.97 (0.92, 1.03)	0.65
	Ever	0.82 (0.79, 0.85)		0.83 (0.81, 0.84)		0.99 (0.95, 1.03)	
Decreased eGFRcys (<90 ml/min/1.73m^2) versus normal or high (≥90)	Never	1.48 (1.25, 1.75)	0.27	1.45 (1.26, 1.67)	0.80	1.02 (0.82, 1.27)	0.52
	Ever	1.31 (1.15, 1.50)		1.48 (1.36, 1.61)		0.89 (0.76, 1.04)	
C-reactive protein, per 1 mg/L	Never	1.17 (1.12, 1.22)	0.40	1.12 (1.07, 1.18)	0.51	1.04 (0.97, 1.11)	0.48
	Ever	1.14 (1.10, 1.18)		1.14 (1.12, 1.17)		1.00 (0.96, 1.04)	
Alcohol drinker status			0.17		0.17		0.74
Previous versus never	Never	1.30 (0.96, 1.77)	0.22	1.35 (0.97, 1.89)	0.16	0.96 (0.61, 1.51)	0.93
	Ever	1.01 (0.78, 1.32)		1.00 (0.77, 1.29)		1.02 (0.71, 1.47)	
Current versus never	Never	0.65 (0.54, 0.78)	0.44	0.77 (0.61, 0.98)	0.38	0.84 (0.62, 1.14)	0.84
	Ever	0.58 (0.47, 0.72)		0.67 (0.53, 0.84)		0.87 (0.64, 1.19)	
Frequency of alcohol consumption[c]			0.02		0.05		0.98
Special occasions only versus never	Never	0.94 (0.76, 1.16)	0.31	1.16 (0.88, 1.52)	0.15	0.81 (0.57, 1.15)	0.74
	Ever	0.80 (0.63, 1.00)		0.88 (0.69, 1.13)		0.91 (0.65, 1.27)	
One to three times a month versus never	Never	0.78 (0.61, 0.99)	0.13	0.90 (0.67, 1.20)	0.31	0.87 (0.60, 1.26)	0.64
	Ever	0.60 (0.47, 0.77)		0.74 (0.57, 0.95)		0.81 (0.57, 1.16)	
Once or twice a week versus never	Never	0.57 (0.46, 0.72)	0.63	0.81 (0.63, 1.04)	0.35	0.71 (0.51, 1.00)	0.98
	Ever	0.53 (0.42, 0.67)		0.68 (0.54, 0.87)		0.78 (0.56, 1.08)	
Three or four times a week versus never	Never	0.48 (0.37, 0.61)	0.92	0.60 (0.46, 0.78)	0.74	0.80(0.56, 1.15)	0.80
	Ever	0.47 (0.37, 0.60)		0.57 (0.45, 0.72)		0.83 (0.59, 1.17)	
Daily or almost daily versus never	Never	0.46 (0.35, 0.60)	0.38	0.70 (0.54, 0.91)	0.73	0.65 (0.44, 0.95)	0.59
	Ever	0.54 (0.43, 0.68)		0.66 (0.52, 0.84)		0.81 (0.59, 1.13)	

AHA denotes American Heart Association, CI confidence interval, eGFR estimated glomerular filtration rate, HDL high-density lipoprotein, HR hazard ratio, LDL low-density lipoprotein

[a]Defined as diagnosis before the age of 30 and receiving insulin treatment.

[b]Socioeconomic status was determined using the Townsend Deprivation Index and grouped into five groups based on the cut-offs for the UK national equal fifths, with the 1st group containing the least socially deprived and the 5th group the most deprived.

[c]Frequency of alcohol consumption was only collected from current alcohol drinkers.
