# Supplementary material for: Sex differences in risk factors for incident peripheral artery disease hospitalisation or death: Cohort study of UK Biobank participants
Source: PLoS One. 2023 Oct 18;18(10):e0292083. doi: 10.1371/journal.pone.0292083 (PMC10584119; doi:10.1371/journal.pone.0292083)
Supplement: S12 Table — (PDF) [file pone.0292083.s018.pdf]

S12 Table. Sex-specific multivariable-adjusted hazard ratios and women-to-men ratio of hazard ratios for risk factors by presence or absence of diabetes.

Risk factors (higher continuous variables or by category for categorical variables)	Diabetes (type 1 or 2)	Women		Men		Women-to-men	
		**HR (95% CI)**	**P value**	**HR (95% CI)**	**P value**	**Ratio of HRs (95% CI)**	**P value**
Systolic blood pressure, per 10 mmHg	No	1.11 (1.08, 1.14)	0.09	1.08 (1.06, 1.10)	0.65	1.03 (1.00, 1.06)	0.14
	Yes	1.05 (0.99, 1.11)		1.07 (1.03, 1.10)		0.98 (0.92, 1.05)	
Diastolic blood pressure, per 5 mmHg	No	1.00 (0.98, 1.02)	<0.001	0.98 (0.97, 1.00)	<0.001	1.02 (0.99, 1.04)	0.41
	Yes	0.88 (0.84, 0.94)		0.90 (0.88, 0.93)		0.98 (0.92, 1.04)	
Pulse pressure, per 5 mmHg	No	1.09 (1.07, 1.10)	0.80	1.07 (1.06, 1.09)	0.17	1.01 (0.99, 1.03)	0.20
	Yes	1.08 (1.05, 1.12)		1.09 (1.07, 1.11)		0.99 (0.95, 1.03)	
AHA hypertension categories			0.10		0.34		0.31
Elevated versus normal	No	0.98 (0.81, 1.19)	0.46	1.05 (0.88, 1.24)	0.81	0.94 (0.73, 1.21)	0.65
	Yes	1.18 (0.76, 1.82)		1.09 (0.82, 1.44)		1.08 (0.64, 1.81)	
Stage 1 hypertension versus normal	No	1.07 (0.91, 1.26)	0.68	1.00 (0.86, 1.16)	0.91	1.07 (0.86, 1.34)	0.62
	Yes	0.98 (0.66, 1.45)		1.02 (0.79, 1.31)		0.96 (0.60, 1.54)	
Stage 2 hypertension versus normal	No	1.41 (1.21, 1.63)	0.36	1.17 (1.02, 1.34)	0.67	1.20 (0.98, 1.47)	0.51
	Yes	1.17 (0.81, 1.68)		1.10 (0.87, 1.40)		1.06 (0.68, 1.64)	
Smoking status			<0.001		<0.001		0.06
Former versus never smokers	No	1.60 (1.45, 1.77)	0.40	2.05 (1.89, 2.23)	<0.001	0.78 (0.68, 0.89)	0.005
	Yes	1.77 (1.43, 2.20)		1.51 (1.32, 1.72)		1.17 (0.91, 1.51)	
Current versus never smokers	No	5.76 (5.17, 6.41)	<0.001	5.26 (4.80, 5.76)	<0.001	1.09 (0.95, 1.26)	0.13
	Yes	2.94 (2.22, 3.90)		2.17 (1.83, 2.57)		1.36 (0.98, 1.88)	
Former versus current smokers	No	0.28 (0.25, 0.31)	<0.001	0.39 (0.36, 0.42)	<0.001	0.71 (0.62, 0.82)	0.46
	Yes	0.60 (0.45, 0.80)		0.68 (0.59, 0.80)		0.88 (0.63, 1.22)	
Current versus non-current smokers	No	3.60 (3.23, 4.01)	<0.001	2.57 (2.38, 2.78)	<0.001	1.40 (1.22, 1.60)	0.68
	Yes	1.66 (1.25, 2.22)		1.46 (1.25, 1.71)		1.14 (0.82, 1.58)	
By smoking intensity[a]			<0.001		<0.001		0.44
≤9 cigarettes per day versus never	No	4.06 (3.22, 5.12)	0.18	4.07 (3.22, 5.14)	<0.001	1.00 (0.72, 1.39)	0.12
	Yes	2.43 (1.19, 4.96)		1.10 (0.54, 2.21)		2.22 (0.82, 6.04)	
10-19 cigarettes per day versus never	No	6.68 (5.78, 7.73)	<0.001	6.62 (5.80, 7.56)	<0.001	1.01 (0.83, 1.23)	0.61

	Yes	2.61 (1.66, 4.09)		2.33 (1.76, 3.09)		1.12 (0.66, 1.90)	
≥20 cigarettes per day versus never	No	8.92 (7.61, 10.46)	<0.001	8.20 (7.26, 9.28)	<0.001	1.09 (0.89, 1.33)	0.32
	Yes	4.04 (2.73, 5.99)		3.09 (2.44, 3.91)		1.31 (0.83, 2.07)	
Cholesterol, per 1 mmol/L							
Total cholesterol	No	0.98 (0.94, 1.02)	0.10	1.01 (0.97, 1.04)	0.64	0.97 (0.92, 1.03)	0.57
	Yes	1.08 (0.97, 1.20)		1.03 (0.96, 1.09)		1.05 (0.93, 1.19)	
HDL-C	No	0.64 (0.55, 0.74)	0.20	0.83 (0.73, 0.93)	0.78	0.77 (0.64, 0.93)	0.39
	Yes	0.82 (0.58, 1.17)		0.79 (0.63, 1.00)		1.03 (0.68, 1.58)	
LDL-C	No	1.02 (0.97, 1.08)	0.21	1.02 (0.97, 1.07)	0.72	1.00 (0.93, 1.08)	0.70
	Yes	1.13 (0.98, 1.30)		1.04 (0.95, 1.13)		1.08 (0.92, 1.28)	
Elevated (≥6.2 mmol/L) versus normal total cholesterol	No	1.01 (0.92, 1.11)	0.07	1.02 (0.94, 1.12)	0.62	0.99 (0.86, 1.12)	0.51
	Yes	1.42 (1.00, 2.02)		1.11 (0.82, 1.49)		1.28 (0.81, 2.03)	
HDL-C categories (versus >1.55 and ≤2.07)			0.04		0.004		0.88
≤1.03	No	1.54 (1.29, 1.83)	0.52	1.25 (1.15, 1.36)	0.83	1.23 (1.01, 1.49)	0.65
	Yes	1.39 (1.07, 1.80)		1.28 (1.12, 1.45)		1.09 (0.81, 1.46)	
>1.03 and ≤1.55	No	0.83 (0.74, 0.92)	0.23	0.88 (0.79, 0.99)	0.03	0.93 (0.80, 1.09)	0.54
	Yes	1.02 (0.73, 1.43)		1.22 (0.93, 1.60)		0.84 (0.54, 1.29)	
>2.07	No	0.76 (0.63, 0.91)	0.32	1.48 (1.19, 1.84)	0.2	0.51 (0.39, 0.68)	0.95
	Yes	1.06 (0.56, 2.02)		2.11 (1.29, 3.43)		0.50 (0.22, 1.13)	
Body mass index, per 5 kg/m^2	No	1.16 (1.11, 1.21)	0.63	1.18 (1.14, 1.23)	0.58	0.98 (0.93, 1.03)	0.24
	Yes	1.18 (1.10, 1.27)		1.16 (1.10, 1.22)		1.02 (0.93, 1.11)	
Body mass index (kg/m^2) categories			0.75		0.30		1.00
Underweight (<18.5) versus healthy weight (18.5-24.9)	No	2.00 (1.41, 2.82)	0.97	1.86 (1.18, 2.93)	0.89	1.07 (0.61, 1.90)	0.95
	Yes	Infinite coefficient		1.65 (0.41, 6.69)		Infinite coefficient	
Overweight (25-29.9) versus healthy weight (18.5-24.9)	No	1.14 (1.03, 1.26)	0.34	0.92 (0.84, 0.99)	0.94	1.25 (1.10, 1.42)	0.43
	Yes	0.95 (0.65, 1.37)		0.92 (0.75, 1.14)		1.03 (0.67, 1.57)	
Obese (≥30) versus healthy weight (18.5-24.9)	No	1.42 (1.27, 1.58)	0.63	1.35 (1.23, 1.47)	0.52	1.05 (0.91, 1.21)	0.86
	Yes	1.30 (0.93, 1.81)		1.26 (1.03, 1.53)		1.03 (0.70, 1.52)	
Waist circumference, per 10 cm	No	1.21 (1.17, 1.25)	0.23	1.17 (1.14, 1.21)	0.70	1.03 (0.99, 1.07)	0.23
	Yes	1.26 (1.18, 1.35)		1.18 (1.14, 1.23)		1.07 (0.99, 1.15)	
Waist-to-hip ratio, per 0.1	No	1.37 (1.32, 1.42)	0.12	1.44 (1.37, 1.51)	0.63	0.95 (0.90, 1.01)	0.23
	Yes	1.51 (1.33, 1.71)		1.47 (1.35, 1.59)		1.03 (0.89, 1.20)	

Waist-to-height ratio, per 0.1	No	1.36 (1.29, 1.43)	0.31	1.35 (1.29, 1.42)	0.65	1.00 (0.94, 1.08)	0.14
	Yes	1.44 (1.30, 1.60)		1.32 (1.23, 1.42)		1.09 (0.96, 1.24)	
History of stroke versus no	No	2.77 (2.23, 3.44)	0.23	2.79 (2.44, 3.18)	<0.001	0.99 (0.77, 1.28)	0.002
	Yes	3.45 (2.54, 4.69)		1.70 (1.38, 2.10)		2.03 (1.40, 2.93)	
History of myocardial infarction versus no	No	4.66 (3.83, 5.66)	0.005	2.94 (2.66, 3.24)	0.004	1.59 (1.27, 1.97)	0.15
	Yes	2.68 (1.94, 3.71)		2.29 (2.00, 2.62)		1.17 (0.83, 1.66)	
Socioeconomic status[b]			0.13		0.62		0.08
2nd versus 1st	No	1.00 (0.88, 1.14)	0.45	1.11 (1.01, 1.23)	0.44	0.90 (0.77, 1.06)	0.77
	Yes	1.16 (0.81, 1.68)		1.20 (1.01, 1.44)		0.97 (0.64, 1.45)	
3rd versus 1st	No	1.16 (1.01, 1.33)	0.18	1.33 (1.20, 1.48)	0.003	0.87 (0.73, 1.03)	0.006
	Yes	1.51 (1.06, 2.15)		0.93 (0.75, 1.15)		1.62 (1.07, 2.44)	
4th versus 1st	No	1.25 (1.09, 1.44)	0.21	1.29 (1.16, 1.44)	0.54	0.97 (0.82, 1.16)	0.15
	Yes	1.59 (1.13, 2.23)		1.20 (0.99, 1.46)		1.32 (0.89, 1.95)	
5th versus 1st	No	1.45 (1.27, 1.65)	0.16	1.72 (1.56, 1.90)	0.82	0.84 (0.72, 0.99)	0.13
	Yes	1.84 (1.35, 2.52)		1.68 (1.43, 1.97)		1.10 (0.77, 1.56)	
eGFRcys, per 10 ml/min/1.73m^2	No	0.81 (0.79, 0.84)	0.02	0.83 (0.81, 0.85)	0.003	0.97 (0.94, 1.01)	0.76
	Yes	0.75 (0.71, 0.80)		0.78 (0.76, 0.81)		0.95 (0.89, 1.02)	
Decreased eGFRcys (<90 ml/min/1.73m^2) versus normal or high (≥90)	No	1.34 (1.20, 1.50)	0.11	1.41 (1.30, 1.54)	0.05	0.95 (0.83, 1.09)	0.80
	Yes	1.73 (1.28, 2.33)		1.67 (1.44, 1.94)		1.03 (0.74, 1.44)	
C-reactive protein, per 1 mg/L	No	1.16 (1.12, 1.19)	0.45	1.14 (1.11, 1.16)	0.94	1.02 (0.98, 1.06)	0.76
	Yes	1.13 (1.06, 1.20)		1.14 (1.09, 1.19)		0.99 (0.92, 1.07)	
Alcohol drinker status			0.67		0.67		0.72
Previous versus never	No	1.17 (0.94, 1.46)	0.95	1.10 (0.85, 1.42)	0.15	1.06 (0.76, 1.49)	0.61
	Yes	1.18 (0.81, 1.73)		1.48 (1.08, 2.02)		0.80 (0.49, 1.30)	
Current versus never	No	0.71 (0.60, 0.83)	0.80	0.84 (0.68, 1.04)	0.36	0.84 (0.64, 1.10)	0.92
	Yes	0.74 (0.56, 0.98)		0.98 (0.76, 1.28)		0.75 (0.51, 1.11)	
Frequency of alcohol consumption[c]			0.73		0.82		0.59
Special occasions only versus never	No	0.94 (0.78, 1.12)	0.60	1.06 (0.84, 1.35)	0.88	0.88 (0.65, 1.19)	0.91
	Yes	0.85 (0.62, 1.16)		1.10 (0.81, 1.47)		0.78 (0.51, 1.20)	
One to three times a month versus never	No	0.75 (0.62, 0.92)	0.86	0.86 (0.67, 1.09)	0.28	0.88 (0.64, 1.20)	0.80
	Yes	0.78 (0.54, 1.14)		1.06 (0.78, 1.44)		0.74 (0.46, 1.20)	
Once or twice a week versus never	No	0.66 (0.55, 0.79)	0.29	0.84 (0.67, 1.05)	0.35	0.78 (0.59, 1.05)	0.29

	Yes	0.53 (0.36, 0.77)		1.00 (0.75, 1.32)		0.53 (0.33, 0.85)	
Three or four times a week versus never	No	0.57 (0.47, 0.70)	0.75	0.73 (0.58, 0.91)	0.54	0.79 (0.59, 1.06)	0.78
	Yes	0.53 (0.33, 0.85)		0.82 (0.61, 1.10)		0.65 (0.37, 1.13)	
Daily or almost daily versus never	No	0.62 (0.51, 0.75)	0.23	0.86 (0.69, 1.07)	0.47	0.72 (0.54, 0.97)	0.34
	Yes	0.83 (0.54, 1.27)		0.98 (0.74, 1.32)		0.84 (0.50, 1.41)	

AHA denotes American Heart Association, CI confidence interval, eGFR estimated glomerular filtration rate, HDL high-density lipoprotein, HR hazard ratio, LDL low-density lipoprotein

[a]Smoking intensity was only collected from current smokers.

[b]Socioeconomic status was determined using the Townsend Deprivation Index and grouped into five groups based on the cut-offs for the UK national equal fifths, with the 1st group containing the least socially deprived and the 5th group the most deprived.

[c]Frequency of alcohol consumption was only collected from current alcohol drinkers.
