# Supplementary material for: Sex differences in risk factors for incident peripheral artery disease hospitalisation or death: Cohort study of UK Biobank participants
Source: PLoS One. 2023 Oct 18;18(10):e0292083. doi: 10.1371/journal.pone.0292083 (PMC10584119; doi:10.1371/journal.pone.0292083)
Supplement: S13 Table — (PDF) [file pone.0292083.s019.pdf]

S13 Table. Sex-specific multivariable-adjusted hazard ratios and women-to-men ratio of hazard ratios for risk factors by socioeconomic status.

Risk factors (higher continuous variables or by category for categorical variables)	SES	Women		Men		Women-to-men	
		HR (95% CI)	P value	HR (95% CI)	P value	Ratio of HRs (95% CI)	P value
Systolic blood pressure, per 10 mmHg	Low	1.12 (1.08, 1.16)	0.16	1.08 (1.05, 1.10)	0.86	1.04 (1.00, 1.08)	0.22
	High	1.09 (1.05, 1.12)		1.07 (1.05, 1.10)		1.01 (0.98, 1.05)	
Diastolic blood pressure, per 5 mmHg	Low	0.98 (0.95, 1.01)	0.65	0.97 (0.95, 0.99)	0.63	1.01 (0.97, 1.05)	0.80
	High	0.99 (0.96, 1.02)		0.96 (0.95, 0.98)		1.02 (0.99, 1.06)	
Pulse pressure, per 5 mmHg	Low	1.10 (1.08, 1.12)	0.04	1.08 (1.06, 1.09)	0.99	1.02 (1.00, 1.05)	0.11
	High	1.07 (1.05, 1.09)		1.08 (1.06, 1.09)		1.00 (0.97, 1.02)	
AHA hypertension categories			0.20		0.36		0.06
Elevated versus normal	Low	1.13 (0.87, 1.46)	0.26	1.05 (0.86, 1.29)	0.89	1.08 (0.77, 1.49)	0.33
	High	0.93 (0.73, 1.18)		1.07 (0.87, 1.32)		0.86 (0.63, 1.18)	
Stage 1 hypertension versus normal	Low	1.16 (0.93, 1.45)	0.23	0.98 (0.82, 1.18)	0.68	1.18 (0.89, 1.57)	0.19
	High	0.97 (0.79, 1.19)		1.04 (0.86, 1.25)		0.93 (0.71, 1.23)	
Stage 2 hypertension versus normal	Low	1.53 (1.25, 1.88)	0.11	1.11 (0.94, 1.31)	0.52	1.38 (1.06, 1.80)	0.07
	High	1.23 (1.02, 1.48)		1.20 (1.01, 1.42)		1.02 (0.79, 1.32)	
Smoking status			0.60		0.11		0.20
Former versus never smokers	Low	1.63 (1.41, 1.88)	0.80	1.85 (1.65, 2.06)	0.09	0.88 (0.74, 1.06)	0.22
	High	1.59 (1.42, 1.79)		2.09 (1.91, 2.28)		0.76 (0.66, 0.88)	
Current versus never smokers	Low	4.92 (4.26, 5.68)	0.35	3.99 (3.55, 4.48)	0.06	1.23 (1.03, 1.48)	0.74
	High	5.42 (4.71, 6.24)		4.65 (4.17, 5.19)		1.17 (0.98, 1.39)	
Former versus current smokers	Low	0.33 (0.29, 0.38)	0.25	0.46 (0.41, 0.50)	0.76	0.72 (0.61, 0.86)	0.40
	High	0.29 (0.25, 0.34)		0.45 (0.40, 0.49		0.65 (0.55, 0.78)	
Current versus non-current smokers	Low	3.91 (3.46, 4.42)	0.13	2.78 (2.54, 3.04)	0.15	1.40 (1.21, 1.64)	0.60
	High	4.49 (3.95, 5.12)		3.06 (2.79, 3.36)		1.47 (1.25, 1.72)	
By smoking intensity[a]			0.01		<0.001		0.57
≤9 cigarettes per day versus never	Low	3.17 (2.29, 4.38)	0.27	2.78 (2.06, 3.74)	0.36	1.14 (0.73, 1.77)	0.83
	High	4.07 (3.01, 5.49)		3.41 (2.47, 4.70)		1.19 (0.77, 1.85)	
10-19 cigarettes per day versus never	Low	5.02 (4.14, 6.09)	0.04	4.43 (3.77, 5.22)	0.002	1.13 (0.88, 1.46)	0.77
	High	6.71 (5.52, 8.16)		6.39 (5.40, 7.57)		1.05 (0.81, 1.36)	

≥20 cigarettes per day versus never	Low	7.32 (6.04, 8.88)	0.29	5.62 (4.87, 6.49)	0.002	1.30 (1.02, 1.66)	0.43
	High	8.64 (6.88, 10.86)		7.94 (6.75, 9.33)		1.09 (0.82, 1.44)	
Diabetes			0.46		0.63		0.43
Type 1 diabetes versus no diabetes	Low	6.16 (3.53, 10.76)	0.75	4.06 (2.60, 6.35)	0.46	1.52 (0.74, 3.10)	0.42
	High	5.42 (2.97, 9.91)		5.11 (3.43, 7.61)		1.06 (0.52, 2.19)	
Type 2 diabetes[b] versus no diabetes	Low	2.10 (1.75, 2.52)	0.44	2.34 (2.08, 2.62)	0.59	0.90 (0.72, 1.12)	0.44
	High	1.89 (1.56, 2.30)		2.24 (2.01, 2.50)		0.84 (0.68, 1.05)	
Cholesterol, per 1 mmol/L							
Total cholesterol	Low	1.00 (0.94, 1.06)	0.81	0.97 (0.93, 1.02)	0.04	1.02 (0.95, 1.10)	0.55
	High	0.99 (0.94, 1.04)		1.04 (1.00, 1.08)		0.95 (0.89, 1.02)	
HDL-C	Low	0.60 (0.49, 0.73)	0.20	0.90 (0.77, 1.06)	0.10	0.66 (0.51, 0.85)	0.01
	High	0.71 (0.60, 0.84)		0.75 (0.65, 0.87)		0.94 (0.75, 1.19)	
LDL-C	Low	1.06 (0.98, 1.14)	0.44	0.96 (0.90, 1.02)	0.006	1.10 (1.00, 1.22)	0.17
	High	1.01 (0.95, 1.09)		1.07 (1.02, 1.14)		0.94 (0.86, 1.03)	
Elevated (≥6.2 mmol/L) versus normal total cholesterol	Low	1.17 (1.01, 1.35)	0.02	0.94 (0.83, 1.07)	0.11	1.24 (1.03, 1.51)	0.04
	High	0.94 (0.83, 1.07)		1.08 (0.97, 1.21)		0.87 (0.74, 1.03)	
HDL-C categories (versus >1.55 and ≤2.07)			0.84		0.31		0.23
≤1.03	Low	1.49 (1.22, 1.82)	0.77	1.26 (1.13, 1.40)	0.97	1.18 (0.95, 1.48)	0.97
	High	1.56 (1.26, 1.92)		1.26 (1.15, 1.39)		1.23 (0.98, 1.55)	
>1.03 and ≤1.55	Low	0.83 (0.71, 0.97)	0.73	0.91 (0.77, 1.07)	0.68	0.91 (0.73, 1.15)	0.84
	High	0.86 (0.75, 0.98)		0.95 (0.83, 1.09)		0.91 (0.75, 1.10)	
>2.07	Low	0.75 (0.56, 0.99)	0.64	1.95 (1.50, 2.53)	0.03	0.38 (0.26, 0.56)	0.03
	High	0.82 (0.65, 1.02)		1.24 (0.91, 1.68)		0.66 (0.45, 0.96)	
Body mass index, per 5 kg/m^2	Low	1.23 (1.18, 1.29)	0.11	1.26 (1.21, 1.31)	<0.001	0.98 (0.92, 1.04)	0.19
	High	1.30 (1.24, 1.37)		1.42 (1.36, 1.48)		0.92 (0.86, 0.98)	
Body mass index (kg/m^2) categories			0.12		0.04		0.90
Underweight (<18.5) versus healthy weight (18.5-24.9)	Low	1.73 (1.06, 2.82)	0.61	1.57 (0.92, 2.67)	0.43	1.10 (0.54, 2.27)	0.76
	High	2.08 (1.28, 3.38)		2.27 (1.08, 4.79)		0.92 (0.38, 2.23)	
Overweight (25-29.9) versus healthy weight (18.5-24.9)	Low	1.09 (0.93, 1.26)	0.17	0.98 (0.87, 1.10)	0.98	1.11 (0.92, 1.34)	0.29
	High	1.25 (1.10, 1.41)		0.98 (0.88, 1.08)		1.27 (1.08, 1.50)	
	Low	1.55 (1.34, 1.79)	0.07	1.52 (1.35, 1.71)	0.03	1.02 (0.85, 1.23)	0.98

Obese (≥30) versus healthy weight (18.5-24.9)	High	1.86 (1.62, 2.12)		1.82 (1.64, 2.02)		1.02 (0.86, 1.21)	
Waist circumference, per 10 cm	Low	1.28 (1.23, 1.33)	0.11	1.26 (1.22, 1.30)	0.02	1.01 (0.96, 1.07)	0.83
	High	1.34 (1.29, 1.39)		1.33 (1.29, 1.37)		1.01 (0.96, 1.06)	
Waist-to-hip ratio, per 0.1	Low	1.69 (1.57, 1.82)	<0.001	1.59 (1.51, 1.68)	0.14	1.06 (0.97, 1.16)	<0.001
	High	1.39 (1.34, 1.43)		1.68 (1.60, 1.77)		0.82 (0.78, 0.87)	
Waist-to-height ratio, per 0.1	Low	1.50 (1.41, 1.60)	0.20	1.52 (1.44, 1.61)	0.02	0.99 (0.91, 1.07)	0.55
	High	1.59 (1.49, 1.69)		1.67 (1.58, 1.77)		0.95 (0.87, 1.03)	
History of stroke versus no	Low	3.35 (2.63, 4.26)	0.97	2.51 (2.14, 2.95)	0.33	1.33 (1.00, 1.78)	0.61
	High	3.33 (2.58, 4.30)		2.82 (2.41, 3.30)		1.18 (0.88, 1.60)	
History of myocardial infarction versus no	Low	4.25 (3.37, 5.35)	0.14	3.11 (2.77, 3.49)	0.77	1.37 (1.06, 1.77)	0.21
	High	5.47 (4.31, 6.94)		3.19 (2.86, 3.56)		1.71 (1.32, 2.23)	
eGFRcys, per 10 ml/min/1.73m^2	Low	0.80 (0.77, 0.83)	0.98	0.83 (0.80, 0.85)	0.26	0.97 (0.92, 1.02)	0.36
	High	0.80 (0.77, 0.83)		0.81 (0.79, 0.83)		0.99 (0.94, 1.04)	
Decreased eGFRcys (<90 ml/min/1.73m^2) versus normal or high (≥90)	Low	1.47 (1.25, 1.72)	0.29	1.56 (1.39, 1.74)	0.18	0.94 (0.77, 1.15)	0.72
	High	1.31 (1.14, 1.50)		1.41 (1.28, 1.55)		0.93 (0.79, 1.10)	
C-reactive protein, per 1 mg/L	Low	1.17 (1.13, 1.21)	0.19	1.15 (1.11, 1.18)	0.56	1.02 (0.97, 1.07)	0.27
	High	1.13 (1.08, 1.17)		1.13 (1.10, 1.17)		1.00 (0.95, 1.05)	
Alcohol drinker status			0.10		0.51		0.22
Previous versus never	Low	1.03 (0.80, 1.33)	0.31	1.13 (0.88, 1.46)	0.88	0.91 (0.64, 1.31)	0.58
	High	1.27 (0.94, 1.70)		1.10 (0.81, 1.49)		1.15 (0.75, 1.76)	
Current versus never	Low	0.56 (0.47, 0.68)	0.10	0.76 (0.61, 0.95)	0.54	0.74 (0.55, 0.98)	0.24
	High	0.71 (0.58, 0.88)		0.69 (0.54, 0.88)		1.04 (0.75, 1.43)	
Frequency of alcohol consumption[c]			0.98		0.42		0.41
Special occasions only versus never	Low	0.73 (0.59, 0.90)	0.02	1.02 (0.80, 1.30)	0.71	0.72 (0.52, 0.99)	0.11
	High	1.06 (0.84, 1.34)		0.95 (0.72, 1.24)		1.12 (0.78, 1.60)	
One to three times a month versus never	Low	0.57 (0.44, 0.72)	0.03	0.86 (0.67, 1.11)	0.47	0.66 (0.46, 0.93)	0.08
	High	0.82 (0.64, 1.06)		0.75 (0.57, 0.99)		1.10 (0.76, 1.59)	
Once or twice a week versus never	Low	0.51 (0.41, 0.63)	0.16	0.76 (0.61, 0.96)	0.73	0.66 (0.48, 0.91)	0.34
	High	0.64 (0.50, 0.80)		0.72 (0.56, 0.93)		0.89 (0.63, 1.25)	
Three or four times a week versus never	Low	0.45 (0.35, 0.58)	0.27	0.63 (0.50, 0.80)	0.53	0.71 (0.50, 1.00)	0.36
	High	0.55 (0.43, 0.70)		0.57 (0.44, 0.73)		0.96 (0.68, 1.37)	

Daily or almost daily versus never	Low	0.48 (0.38, 0.62)	0.19	0.71 (0.56, 0.89)	0.89	0.69 (0.49, 0.96)	0.50
	High	0.61 (0.48, 0.78)		0.69 (0.53, 0.89)		0.89 (0.62, 1.26)	

AHA denotes American Heart Association, CI confidence interval, eGFRcys estimated Glomerular Filtration Rate calculated using cystatin C, HDL high-density lipoprotein, HR hazard ratio, LDL low-density lipoprotein, SES socioeconomic status.

[a]Smoking intensity was only collected from current smokers.

[b]Defined as diagnosis before the age of 30 years old and receiving insulin treatment.

[c]Frequency of alcohol consumption was only collected from current alcohol drinkers.
