# Supplementary material for: Sex differences in risk factors for incident peripheral artery disease hospitalisation or death: Cohort study of UK Biobank participants
Source: PLoS One. 2023 Oct 18;18(10):e0292083. doi: 10.1371/journal.pone.0292083 (PMC10584119; doi:10.1371/journal.pone.0292083)
Supplement: S14 Table — (PDF) [file pone.0292083.s020.pdf]

S14 Table. Sex-specific multivariable-adjusted hazard ratios and women-to-men ratio of hazard ratios for blood pressure by use of antihypertensive medication.

Blood pressure (higher continuous variables or by category for categorical variables)	Use of antihypertensive medication	Women		Men		Women-to-men	
		HR (95% CI)	P value	HR (95% CI)	P value	Ratio of HRs (95% CI)	P value
Systolic blood pressure, per 10 mmHg	No	1.10 (1.07, 1.13)	0.67	1.10 (1.07, 1.12)	0.02	1.01 (0.97, 1.04)	0.40
	Yes	1.09 (1.06, 1.13)		1.05 (1.03, 1.08)		1.04 (1.00, 1.08)	
Diastolic blood pressure, per 5 mmHg	No	1.01 (0.98, 1.03)	0.01	1.03 (1.01, 1.06)	<0.001	0.97 (0.94, 1.01)	0.12
	Yes	0.95 (0.92, 0.98)		0.91 (0.89, 0.93)		1.04 (1.00, 1.09)	
Pulse pressure, per 5 mmHg	No	1.09 (1.07, 1.11)	0.93	1.07 (1.05, 1.09)	0.29	1.01 (0.99, 1.04)	0.63
	Yes	1.08 (1.06, 1.11)		1.08 (1.07, 1.10)		1.00 (0.98, 1.02)	
AHA hypertension categories			0.04		<0.001		0.93
Elevated versus normal	No	0.92 (0.74, 1.14)	0.24	1.18 (0.96, 1.46)	0.15	0.78 (0.57, 1.05)	0.11
	Yes	1.14 (0.84, 1.54)		0.95 (0.78, 1.17)		1.20 (0.83, 1.72)	
Stage 1 hypertension versus normal	No	1.08 (0.90, 1.30)	0.37	1.11 (0.92, 1.34)	0.12	0.97 (0.75, 1.26)	0.83
	Yes	0.93 (0.71, 1.22)		0.91 (0.76, 1.08)		1.03 (0.74, 1.42)	
Stage 2 hypertension versus normal	No	1.44 (1.22, 1.70)	0.18	1.41 (1.18, 1.68)	0.001	1.02 (0.80, 1.30)	0.65
	Yes	1.17 (0.91, 1.50)		0.95 (0.80, 1.12)		1.24 (0.92, 1.66)	

AHA denotes American Heart Association, CI confidence interval, HR hazard ratio.
