# Supplementary material for: Sex differences in risk factors for incident peripheral artery disease hospitalisation or death: Cohort study of UK Biobank participants
Source: PLoS One. 2023 Oct 18;18(10):e0292083. doi: 10.1371/journal.pone.0292083 (PMC10584119; doi:10.1371/journal.pone.0292083)
Supplement: S15 Table — (PDF) [file pone.0292083.s021.pdf]

S15 Table. Sex-specific multivariable-adjusted hazard ratios and women-to-men ratio of hazard ratios for cholesterol measures by use of lipid lowering medication.

**Cholesterol measures (per 1 mmol/L higher or by category)**	**Use of lipid lowering medication**	**Women**		**Men**		**Women-to-men**	
		**HR (95% CI)**	**P value**	**HR (95% CI)**	**P value**	**Ratio of HRs (95% CI)**	**P value**
Total cholesterol	No	1.00 (0.95, 1.05)	0.53	0.99 (0.95, 1.03)	0.08	1.01 (0.95, 1.08)	0.85
	Yes	0.97 (0.90, 1.04)		1.04 (1.00, 1.09)		0.93 (0.86, 1.01)	
High-density lipoprotein cholesterol	No	0.64 (0.54, 0.75)	0.61	0.84 (0.72, 0.98)	0.90	0.76 (0.61, 0.96)	0.79
	Yes	0.69 (0.55, 0.85)		0.82 (0.70, 0.96)		0.84 (0.64, 1.09)	
Low-density lipoprotein cholesterol	No	1.04 (0.98, 1.11)	0.52	0.98 (0.93, 1.03)	0.02	1.06 (0.98, 1.15)	0.86
	Yes	1.00 (0.91, 1.10)		1.08 (1.02, 1.15		0.93 (0.83, 1.04)	
Elevated (≥6.2 mmol/L) versus normal total cholesterol	No	1.01 (0.91, 1.12)	0.49	1.00 (0.91, 1.10)	0.30	1.00 (0.87, 1.15)	0.84
	Yes	1.09 (0.88, 1.36)		1.12 (0.93, 1.35)		0.98 (0.73, 1.30)	
HDL-C categories (versus >1.55 and ≤2.07)			0.02		0.77		0.06
≤1.03	No	1.58 (1.31, 1.92)	0.48	1.18 (1.07, 1.29)	0.04	0.34 (1.08, 1.67)	0.09
	Yes	1.43 (1.15, 1.78)		1.36 (1.23, 1.52)		1.05 (0.82, 1.34)	
>1.03 and ≤1.55	No	0.92 (0.77, 1.10)	0.24	0.93 (0.79, 1.09)	0.99	0.99 (0.78, 1.25)	0.32
	Yes	0.81 (0.71, 0.91)		0.94 (0.81, 1.07)		0.86 (0.71, 1.04)	
>2.07	No	0.97 (0.71, 1.34)	0.1	1.54 (1.13, 2.10)	0.81	0.63 (0.40, 0.99)	0.16
	Yes	0.71 (0.58, 0.88)		1.63 (1.26, 2.11)		0.44 (0.31, 0.61)	

CI denotes confidence interval, HR hazard ratio.
